# The N. gonorrhoeae Type IV Pilus Stimulates Mechanosensitive Pathways and Cytoprotection through a *pilT*-Dependent Mechanism

**DOI:** 10.1371/journal.pbio.0030100

**Published:** 2005-03-22

**Authors:** Heather L Howie, Michael Glogauer, Magdalene So

**Affiliations:** **1**Department of Molecular Microbiology and Immunology, Oregon Health and Science UniversityPortland, OregonUnited States of America; **2**Canadian Institutes of Health Research Group in Matrix DynamicsUniversity of Toronto, OntarioCanada; Tufts University School of MedicineUnited States of America

## Abstract

The Neisseria gonorrhoeae type IV pilus is a retractile appendage that can generate forces near 100 pN. We tested the hypothesis that type IV pilus retraction influences epithelial cell gene expression by exerting tension on the host membrane. Wild-type and retraction-defective bacteria altered the expression of an identical set of epithelial cell genes during attachment. Interestingly, pilus retraction, per se, did not regulate novel gene expression but, rather, enhanced the expression of a subset of the infection-regulated genes. This is accomplished through mitogen-activated protein kinase activation and at least one other undefined stress-activated pathway. These results can be reproduced by applying artificial force on the epithelial membrane, using a magnet and magnetic beads. Importantly, this retraction-mediated signaling increases the ability of the cell to withstand apoptotic signals triggered by infection. We conclude that pilus retraction stimulates mechanosensitive pathways that enhance the expression of stress-responsive genes and activate cytoprotective signaling. A model for the role of pilus retraction in influencing host cell survival is presented.

## Abstract 2

Force exerted on the membrane of epithelial cells by bacterial attachment enhances the expression of stress-responsive genes

## Introduction

Many pathogenic and nonpathogenic bacteria produce type IV pili (Tfp), among them, Neisseria gonorrhoeae, *N. meningitidis, Pseudomonas aeruginosa, Legionella pneumophila,* enteropathogenic and enterohemorrhagic *Escherichia coli,* and Vibrio cholerae [1]*.* Tfp are fimbriate organelles that play a crucial role in the interaction of the bacterium with its environment, as evidenced by their requirement for motility [2], biofilm formation [3,4], and horizontal gene transfer [5,6,7]. These appendages also promote bacterial attachment to host cells and contribute to virulence [8,9,10,11,12].

Recent evidence has shown that the gonococcal Tfp can physically retract—a process that underlies twitching motility [13] (i.e., the ability of the bacterium to move on solid surfaces [14]). It is now generally believed that twitching motility occurs via extension, substrate tethering, and retraction of the pilus filament. Two inner membrane/cytoplasmic ATPases, PilF and PilT, take part in these activities. PilF mediates pilus assembly, as *pilF* mutants produce pilin subunits but are not piliated [15]. PilT is involved in pilus disassembly, as *pilT* mutants are piliated but cannot retract their pili [13,16]. Neither mutant is motile.

Pilus retraction allows gonococci to form organized microbial communities on the cell surface and on synthetic substrates (S. Lee and M. S., unpublished data), via both specific and nonspecific interactions. During attachment to host cells, microcolonies stimulate the formation of cortical plaques—structures in the cell cortex containing high concentrations of transmembrane receptors, nonreceptor tyrosine kinases and their anchors, and components of the cortical cytoskeleton [10,17]. Though *pilT* mutants adhere normally to both synthetic surfaces and epithelial cells, they form disordered microcolonies, fail to induce cortical plaques, and are less invasive than their wild-type (wt) parent strain [17].

Retraction of a single gonococcal pilus can exert forces up to 80–100 pN on its substrate [13,18]. Forces of lesser magnitude can elongate the membrane into microvillus-like structures [19,20], promote cytoskeleton rearrangements and protein clustering [21,22], induce calcium fluxes [23,24], and alter gene expression [25,26,27,28]. Pilus retraction has therefore been speculated to induce host cell signaling by exerting mechanical tension on the membrane [17]. Indirect support for a mechanical signaling hypothesis comes from observations that *pilT* mutants, unlike wt piliated strains, can neither trigger cortical plaque formation [10] nor activate PI-3 kinase (S. Lee and M. S., unpublished data), a member of a mechanical stress–activated pathway. Moreover, a *pilT* mutant induces an attenuated calcium flux in epithelial cells, as compared to infection with wt gonococci (P. Ayala and M. S., unpublished data). Here we provide further evidence that pilus retraction acts as a mechanical stimulus by activating mechanical stress–signaling pathways that alter epithelial cell gene expression and generate a cytoprotective environment within the host cell.

## Results

### Pilus Retraction Enhances the Expression of Cell Stress/Survival Genes

**Figure 1:**
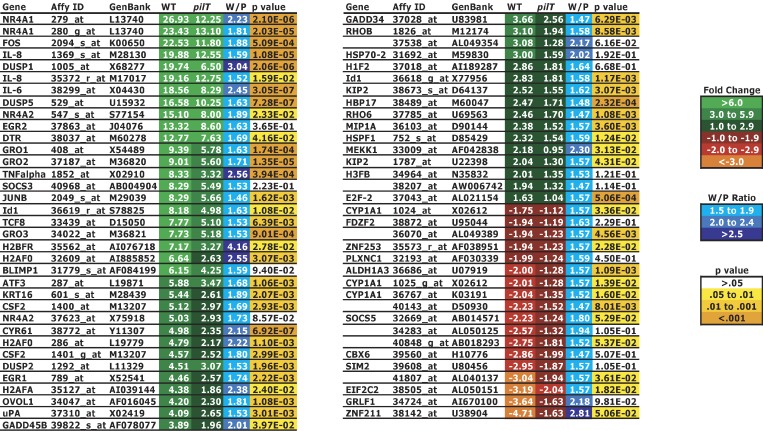
Infection-Regulated, Retraction-Enhanced Epithelial Cell Genes Wt and *pilT* values represent the mean fold-change in the transcript level of each gene in infected cells compared to uninfected cells (*n* = 2). W/P values represent the degree of enhancement of gene expression resulting from pilus retraction and are the result of dividing the wt fold-change value by the *pilT* fold-change value from two independent experiments. The *p*-value for each gene represents the statistical significance of the difference in its expression level (as determined by Cyber-T analysis) between wt and *pilT*. The color code assigned to each gene represents its degree of response to infection as expressed by its fold-change value, W/P, and *p*-value.

We used microarrays to examine the transcriptional profiles of T84 human colorectal epithelial cells infected with retraction-proficient (N400) or retraction-deficient (N400*pilT*) gonococci for 3 h. Infection with N400 or N400*pilT* induced transcriptional changes in the same genes. Contrary to expectations, no genes responded uniquely to infection with either strain. Instead, infection with *pilT* affected the level of expression of a small subset of infection-responsive genes. To segregate the genes responding to pilus retraction, a wt to *pilT* fold-change expression ratio (W/P) was calculated for each infection-regulated gene. This method identified, out of approximately 300 infection-regulated genes, 69 probe sets (representing 52 genes) whose expression appeared to be enhanced by pilus retraction (Figure 1).

**Figure 2:**
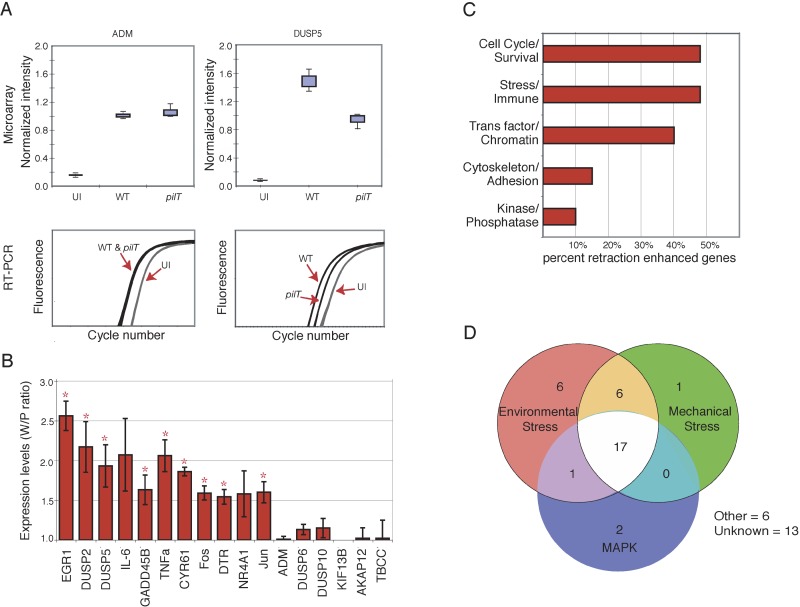
Real-Time Quantitative RT-PCR Verification of Microarray Results and Initial Characterization of Retraction-Enhanced Genes (A) Microarray (top panels) and real-time quantitative RT-PCR (bottom panels) expression profiles of ADM and DUSP5 in uninfected cells (UI), N400-infected cells (WT), and N400*pilT*-infected cells*(pilT)*. Microarray data are shown as box-plots (*n* = 3). RT-PCR data are plotted as triplicate samples from one representative experiment. (B) Real-time quantitative RT-PCR verification of retraction-enhanced expression of selected genes. Data are expressed as average W/P (±SEM, *n* = 3). Genes with a W/P statistically greater than 1.0 (*p* < 0.05) are denoted with an asterisk. (C) Grouping of retraction-enhanced genes according to function, based on published reports (see Table S1). Some genes have multiple functions and thus appear in more than one group. (D) Genes in this study that are known to be induced by environmental stress, mechanical stress, or MAPK signaling (see Table S1).

To confirm the microarray results, real-time quantitative RT-PCR was initially performed on two infection-regulated genes, *DUSP5* and *ADM*. According to our microarray data, *DUSP5* expression was enhanced by pilus retraction (W/P = 1.63), and *ADM* expression was not (W/P ≈ 1.0). RT-PCR results corroborated the microarray analysis, as *DUSP5* transcript levels were significantly higher in N400-infected cells than N400*pilT*-infected cells, whereas *ADM* transcript levels were similar in both sets of cells (Figure 2A). Ten additional genes predicted to respond to retraction and five additional genes predicted to be not affected by retraction were similarly analyzed by real-time quantitative RT-PCR (Figure 2B). In every case, the presumptive positives yielded W/P ratios of 1.5 or more, whereas the presumptive negatives yielded W/P ratios of approximately 1.0.

The identification of genes whose expression is enhanced by pilus retraction raised the question of whether these genes share a common regulatory pathway or perform similar functions. The majority of genes whose expression is enhanced by retraction are involved in the cell stress response and survival (Figure 2C). Over half of these can be induced by environmental or other cellular stresses, and a striking number can be induced specifically by mechanical stress (Figure 2D). Importantly, the majority of the genes from both groups can also be induced by mitogen-activated protein kinases (MAPKs; Figure 2D) (For literature citations, see Table S1.) These results indicate that pilus retraction may enhance infection-induced gene expression through the MAPK pathway.

### ERK, JNK, and P38 MAPK Are Activated by Infection and Enhanced by Pilus Retraction

The MAPK cascades are well known for their involvement in the stress response, including the response to bacterial infection. Previous studies have shown that JNK is activated in N. gonorrhoeae–infected HeLa, Chang, and phagocytic cells [29,30], and MAPK signaling is induced in conjunctival cells by N. meningitidis (R. Bonnah and M. S., unpublished data). To study the role of MAPK signaling in retraction-enhanced gene expression, we first determined which of these pathways are activated in infected T84 cells.

**Figure 3:**
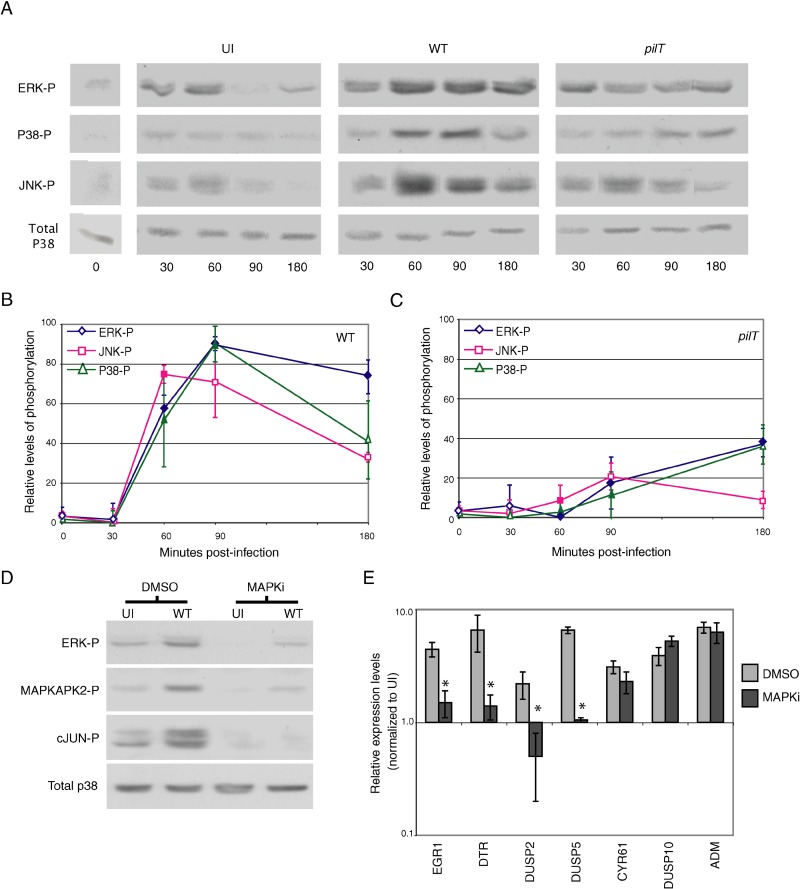
Levels of Activated MAPK in Infected Cells and Their Involvement in Retraction-Enhanced Gene Expression (A) Representative immunoblot showing ERK-p, P38-p, and JNK-p, in uninfected cells before (0 h) and after medium change (UI), or in cells infected with N400 (WT) or N400*pilT (pilT).* Total P38 protein levels in each sample served as the internal control (bottom lanes). (B and C) ERK-p JNK-p, and P38-p levels over time in cells infected with N400 and N400*pilT,* respectively. Immunoblots from (A) were analyzed by densitometry, and levels of activated kinase from infected cells were normalized to that from uninfected cells (UI). Values represent mean normalized protein levels (±SEM, *n* = 2). Solid markers indicate a significant difference between wt and *pilT*-induced MAPK phosphorylation at that time point (*p* < 0.05); thus, ERK-p is significant at 60, 90, and 180 min; JNK-p is significant at 60 min; and P38-p is significant at 60 and 90 min. (D) Representative immunoblot showing ERK-p, MAPKAPK2-p, and c-Jun-p in cells preincubated with vehicle (DMSO) or MAPK inhibitors and infected for 90 min with N400 (WT) or left untreated (UI). Total P38 protein levels in each sample served as the internal control (bottom lanes). (E) Real-time quantitative RT-PCR analysis of the effect of MAPK inhibitors on the expression of retraction-responsive genes. Light bars indicate cells infected with N400 in the presence of vehicle (DMSO); dark bars indicate cells infected with N400 in the presence of MAPK inhibitors. Values represent the fold-change (±SEM, *n* = 2) in transcript levels compared to uninfected, DMSO treated control. A significant difference in expression between the two conditions is denoted by an asterisk (*p* < 0.1).

Compared to resting cells (Figure 3A, left panel), the addition of medium alone slightly increased the levels of ERK-p, JNK-p, and P38-p (Figure 3A, UI), but levels of each phosphorylated kinase returned to baseline after 90 min. Infection with N400 dramatically increased the levels of all three activated kinases by 60 min post-infection (Figure 3A, WT). Densitometric analysis of immunoblots from two independent experiments is shown in Figure 3B. ERK-p levels were elevated throughout the course of infection, with only a slight decrease in phosphorylation visible by 3 h post-infection. In contrast, P38-p and JNK-p levels peaked between 60 and 90 min post-infection and dropped noticeably by 3 h post-infection.

We next examined MAPK phosphorylation in T84 cells infected with N400*pilT* to determine whether kinase activation was influenced by pilus retraction. Low levels of all three activated MAPKs were detected in N400*pilT*-infected cells only after 90 min of infection (Figure 3A, PT). Densitometric analysis of immunoblots from two independent experiments is shown in Figure 3C. Although the kinetics of MAPK activation appear to be different in wt- and *pilT*-infected cells, a firm conclusion cannot be drawn from these results, given the delayed onset of activation and the low levels of phosphorylation of each enzyme. Taken together, these results demonstrate that infection by piliated gonococci activates all three MAPK pathways and that pilus retraction enhances this activation.

### MAPK Signaling Is a Mediator of Retraction-Dependent Enhancement of Gene Expression

We next determined whether MAPK signaling regulates the expression of retraction-enhanced genes. T84 cells were preincubated with vehicle or MAPK inhibitors SB203588, U0126, and SP600125, and assessed for ERK, P38, and JNK activation by immunoblotting for ERK-p, MAPKAPK2-p, and c-Jun-p, respectively. MAPK inhibitors dramatically reduced the levels of all three activated kinases in both uninfected and N400-infected cells (Figure 3D). They also significantly reduced the transcript levels of four of the five retraction-responsive genes in N400-infected cells, as judged by real-time quantitative RT-PCR (Figure 3E). In contrast, the inhibitors did not affect the transcript levels of genes with a W/P of approximately 1.0. Interestingly, *cyr61* expression was unaltered by MAPK inhibitors. This gene was shown by microarray (W/P = 2.15) and RT-PCR analysis (W/P = 1.86) to respond to retraction. These results implicate MAPK signaling in the regulation of some, but not all, retraction-responsive genes. They indicate that other pathways also influence the response of genes to pilus retraction.

### Mechanical Stress Activates MAPK Signaling and Upregulates Retraction-Responsive Genes

**Figure 4:**
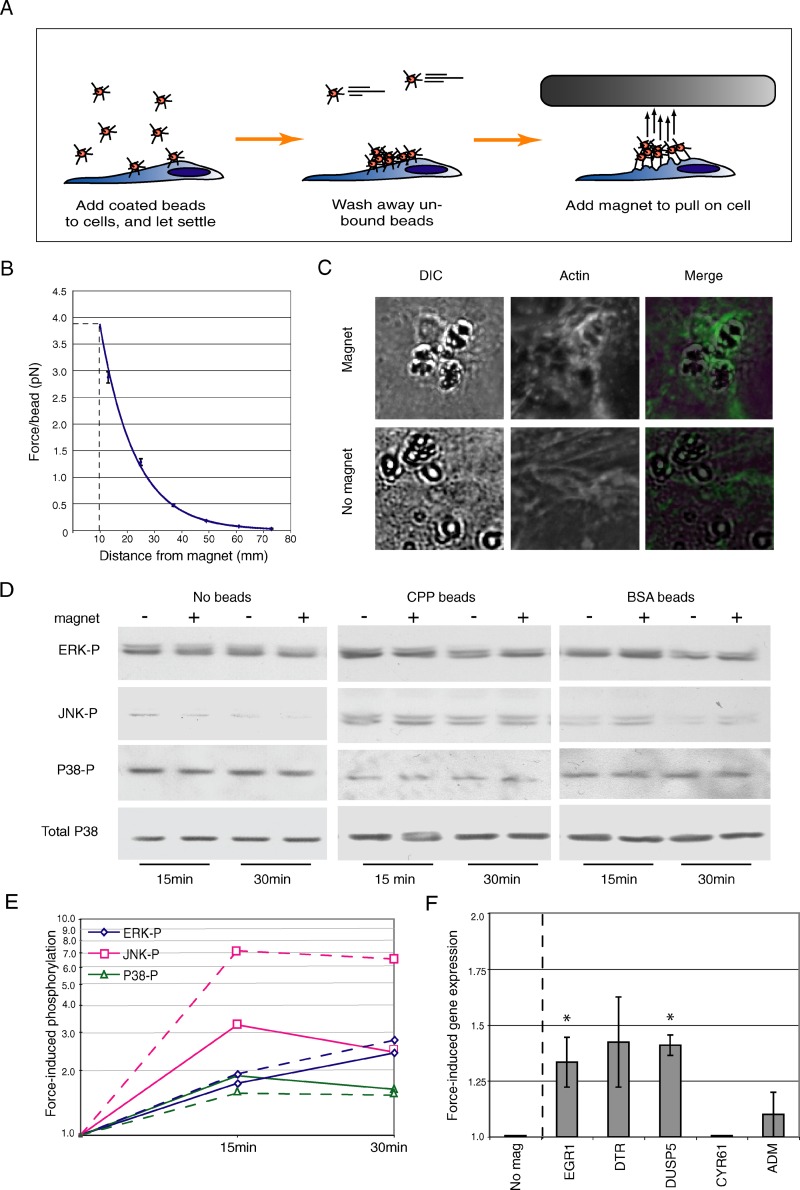
Artificial Force Triggers MAPK Phosphorylation and Induces the Expression of Retraction-Enhanced Genes (A) Representation of the magnet/magnetic bead assay. (B) Average force generated on one bead as a function of magnet distance from the culture dish. Data represent the forces calculated from four identical magnets (±SEM). All subsequent assays were performed using a magnet distance of 10 mm, which corresponds to a force of 4 pN per bead (dotted line). (C) Magnet-induced clustering of actin beneath magnetic beads. CPP-coated beads were seeded onto T84 cells and exposed to the magnet for 1 h (top panels) or left untreated (no magnet, bottom panels). Differential interference contrast images (left panels) reveal the location of the beads; phalloidin staining (middle panels) shows the presence of actin at the same site. Right panels show the two previous images merged. (D) Representative immunoblot of ERK-p, JNK-p, and P38-p in cells seeded with CPP-coated beads, BSA-coated beads, or no beads, and exposed to the magnet for 15 or 30 min. Total P38 protein levels in each sample served as the internal control (bottom panels). (E) Quantitation of ERK-p, JNK-p, and P38-p signals by densitometry from the representative immunoblot shown in (D), normalized to the no-bead control. Solid lines indicate signals from cells exposed to membrane-coated beads; dotted lines indicate signals from cells exposed to BSA-coated beads. (F) Real-time quantitative RT-PCR analysis of the transcript levels of selected genes in cells seeded with CPP beads and exposed to the magnet for 3 h. Data represent the average fold-change (±SEM, *n* = 2) compared to a no-magnet control. A significant difference in expression on force induction is denoted by an asterisk (*p* < 0.1).

A significant number of the retraction-responsive genes are known to be induced specifically by mechanical strain on the cell membrane. Although substantial force is generated by pilus retraction in vitro, this force has not yet been demonstrated to influence host responses to infection. To examine this issue, we determined whether artificial mechanical force on the epithelial cell membrane could mimic retraction-induced MAPK activation and retraction-enhanced gene expression. To generate mechanical stress in a manner similar to that of pilus retraction, a modified magnet-based force assay was used [31]. Magnetic beads were coated with crude pili preparations (CPPs) from piliated gonococci and added to T84 cells (Figure 4A). Within 30 min, small clusters of approximately two to ten beads attached to the cells, with each cell containing two to three clusters of beads (data not shown). Cell monolayers were then placed 10 mm beneath the magnet. At this distance, the magnet generates an upward force of 4 pN per bead (Figure 4B), or approximately 20–100 pN per cell.

T84 cells seeded with CPP-coated beads and exposed to the magnet were first examined for the presence of actin recruitment into cortical plaques (see Introduction). The clustering of actin near these beads would indicate that the magnetic force was sufficient to mimic pilus retraction forces from the bacterial microcolony. In the presence of magnetic force, actin concentrated in the cell cortex around membrane-coated beads (Figure 4C, top panel). In contrast, actin did not cluster with the beads in the absence of the magnet (Figure 4C, bottom panel). Thus, the force generated by this magnet system was sufficient to recruit actin to the site of the attached beads.

We next determined whether magnetic forces applied to CPP-coated beads were sufficient to activate MAPK and alter gene expression. The levels of all three phosphokinases were slightly reduced when the magnetic field was applied to cells incubated with medium alone (Figure 4D, no beads). Levels of each phosphorylated kinase from bead-treated samples (Figure 4D, CPP) were normalized to those from the no-bead samples to account for the effect of the magnet alone on MAPK phosphorylation. Following normalization, increased levels of all three phosphokinases are evident within the short time course (Figure 4E, solid lines).

In parallel experiments, cells were seeded with bovine serum albumin (BSA)-coated beads and exposed to the magnet. Under these conditions, less force was applied to the cells, as fewer bead clusters attached to the cells, and each cluster contained only two to three beads on average (data not shown). Again, levels of each phosphorylated kinase from bead-treated samples (Figure 4D, BSA beads) were normalized to those from the no-bead samples to account for the effect of the magnet alone on MAPK phosphorylation. Despite lower forces, BSA-coated beads also activated ERK, JNK, and P38 (Figure 4E, dashed lines). Interestingly, force-induced activation of both JNK and ERK was higher in cells treated with BSA-coated beads. This can most likely be attributed to the fact that BSA-coated beads, unlike CPP beads, induce no MAPK activation in the absence of force (data not shown). Thus when force-induced MAPK activation is calculated, the CPP-coated beads are normalized to a higher level of “background” activation than are the BSA-coated beads. The observation that force induction via both CPP- and BSA-coated beads can induce these signals strongly indicates that activation of MAPK cascades is, in part, a response to stress forces on the membrane rather than to force mediated through specific adhesin–receptor contacts between the bacterium and the host.

To examine the effect of mechanical stress on gene expression changes, cells seeded with CPP-coated beads were exposed to magnetic force for 3 h, and gene expression levels were analyzed by real-time quantitative RT-PCR. Transcript levels were expressed as the ratio of signals from magnet-stimulated cells to those from cells not subjected to magnetic force. All three “enhanced” genes tested, *EGR1, DTR,* and *DUSP5,* were upregulated in cells exposed to magnetic force (Figure 4F). In contrast, neither *ADM* (W/P ≈ 1.0) nor *cyr61* (which did not respond to MAPK inhibitors; see Figure 3E) was affected by the magnet.

In this and the previous experiment, magnet-induced changes were of lower magnitude than those induced by infection. The most plausible explanation for this difference is that pilus retraction from a microcolony likely generates greater force than a magnet acting on a small cluster of beads. In our magnet assay, an average force of 20–100 pN was placed on each cell. During an infection, each *pilus* can induce this amount of force. Thus, if there are 10–100 bacteria per microcolony, and each bacterium expressed 10 pili (a conservative estimate), pilus retraction from a single microcolony could place forces of 10^4^–10^5^ pN on the cell. Nonetheless, our method of artificial force application did indeed activate all three MAPK cascades and increased the expression level of each gene examined by approximately 1.5-fold. (Note that a minimum 1.5-fold change in expression level was found to accurately identify retraction-responsive genes in the microarray experiment.) Together, these results demonstrate that retraction-enhanced MAPK activation and gene expression changes can be replicated by artificial force.

### Pilus Retraction Mediates Host Cell Cytoprotection

Many of the retraction-responsive genes are known to protect cells from apoptosis and from a variety of cellular stresses. Moreover, prolonged ERK activation accompanied by transient JNK and P38 activation (as observed in a wt infection; see Figure 3A and 3B) is hypothesized to mediate cytoprotection [32,33,34,35]. We therefore investigated whether pilus retraction was involved in determining cell fate by assaying infected cells for cleaved poly(ADP-ribose) polymerase (PARP) and cleaved caspase 8. PARP is a 116-kDa nuclear protein that mediates DNA repair in response to cell stress and is required to maintain cell viability [36,37]. During programmed cell death, the protein is cleaved by caspase 3 or caspase 7, a terminal step in the caspase cascade [38,39]. Caspase 8, however, is an initiator caspase that is upstream of caspase 3, caspase 7, and PARP, and represents an earlier event in the apoptosis cascade. Thus, increased levels of cleaved PARP or caspase 8 indicate that a cell is undergoing apoptosis.

**Figure 5:**
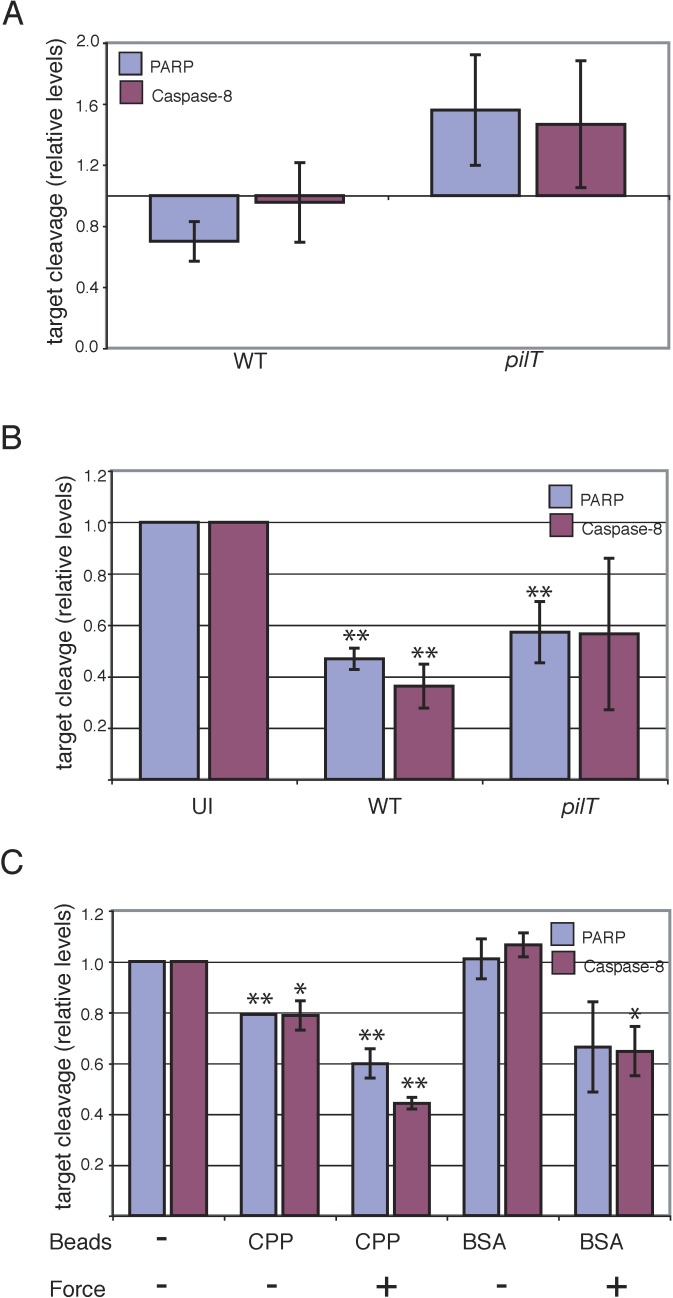
Pilus Retraction during Bacterial Attachment Promotes Host Cell Cytoprotection (A) Levels of cleaved PARP and cleaved caspase 8 in T84 cells infected for 6 h with N400 (WT) or N400*pilT (pilT),* normalized to cleaved PARP or cleaved caspase 8 levels in uninfected cells. (B) Levels of cleaved PARP and cleaved caspase 8 in T84 cells infected with N400 (WT) or N400*pilT (pilT)* or left uninfected (UI) for 4 h, then incubated with STS (1 μM) for an additional 4 h to induce apoptosis. A significant difference from uninfected cells is denoted by two asterisks (*p* < 0.05). (C) Cleaved PARP and cleaved caspase 8 levels in cells exposed to magnetic force. T84 cells were seeded with CPP- or BSA-coated beads and exposed to the magnet for 2 h or were left unexposed, then incubated with STS (1 μM) for an additional 4 h away from the magnet. The cleaved PARP and cleaved caspase 8 level in cells without beads and not exposed to magnetic force is arbitrarily assigned a value of 1.0, and all other treatments are expressed relative to this value. For all experiments, cleaved protein levels were quantified by densitometry of immunoblot signals. Values represent the mean levels of cleaved target (±SEM) from two independent experiments. A significant difference from untreated cells is denoted by two asterisks (*p* < 0.05) or by a single asterisk (*p* < 0.1).

Cells infected with N400 for 6 h contained lower levels of both cleaved PARP and cleaved caspase 8 than did uninfected cells (Figure 5A). In contrast, N400*pilT*-infected cells had higher levels of cleaved PARP and cleaved caspase 8 than did both uninfected and wt-infected cells. These results indicate that piliated gonococci that cannot retract pili induce low levels of programmed cell death in a culture. In contrast, gonococci capable of retracting their pili lower the tendency for cells to enter the apoptosis pathway.

We next determined whether this cytoprotective effect of pilus retraction was sufficient to protect cells from staurosporine (STS)-induced apoptosis. STS is a cell-permeant protein kinase inhibitor that induces apoptosis at micromolar concentrations [40,41]. Infection of urethral epithelium with N. gonorrhoeae was recently reported to protect these cells from STS-induced apoptosis [42]. Both N400 and N400*pilT* infection protected T84 cells from STS-induced apoptosis, as compared to uninfected cells (Figure 5B). However, cleaved PARP and cleaved caspase 8 levels in N400*pilT*-infected cells were higher than in wt-infected cells, indicating that pilus retraction enhances protection from STS-induced apoptosis.

Finally, we examined whether this retraction-enhanced cytoprotection is specifically mediated by mechanical force. In the absence of force, CPP-coated beads provided moderate protection from STS-induced apoptosis, demonstrated by lower cleaved PARP and cleaved caspase 8 levels than the no-bead cell control (Figure 5C). This result is similar to that seen in *pilT*-infected cells and suggests that components in the bacterial membrane are sufficient to protect against STS-induced apoptosis. Cells seeded with CPP-coated beads and exposed to the magnetic field had still lower cleaved PARP and cleaved caspase 8 levels, consistent with data from wt-infected cells (Figure 5B). BSA-coated beads did not protect against STS-induced apoptosis in the absence of magnetic force. However, when force was applied to these cells, the level of cleaved PARP and cleaved caspase 8 was reduced nearly to the value observed for membrane-coated beads in the presence of the magnet. Together, these data indicate that nonspecific membrane tension is capable of protecting the host cell against apoptosis.

## Discussion

Retraction of the N. gonorrhoeae Tfp during bacterial attachment elicits host cell signaling cascades essential for the establishment of intimate attachment and promotion of bacterial invasion [17]. We tested the hypothesis that Tfp retraction induces changes in epithelial cell gene expression during bacterial attachment. Pilus retraction, per se, did not regulate a unique set of genes. Rather, retraction enhanced the expression of a small subset of infection-regulated genes (see Figure 1), many of which are known to respond specifically to mechanical stress and to be induced by the MAPK cascade. We confirmed that wt bacteria activated MAPKs ERK, JNK, and P38 at a higher level than the *pilT* mutant. Moreover, MAPK inhibitors lowered the expression level of all but one retraction-responsive gene selected for further examination (see Figure 3). These results strongly indicate that MAPK signaling plays a major role in the enhancement of gene expression by pilus retraction.

Importantly, artificial force placed on the cell membrane using magnets and magnetic beads can replicate the gene expression changes and MAPK activation observed using wt bacteria, indicating that pilus retraction may induce these events via mechanical force. Although the total force produced by pilus retraction within a bacterial microcolony is not known, we estimate that it is on the order of 10^4^–10^5^ pN, based on 100 pN per retraction event, approximately 10 pili per bacteria, and roughly 10–100 bacteria per microcolony. In comparison, this amount of force is equivalent to that applied to integrin complexes in the periodontal ligament by a human bite [31]. Retraction forces from a microcolony could therefore be physiologically relevant.

We cannot exclude the possibility that pilus retraction enhances these signaling events by mechanisms independent of membrane tension (i.e., through secondary receptor engagement or via an inherent difference in the pilus structure/composition between wt and *pilT* bacteria). Our data with CPP-coated beads strongly argue against these possibilities, however. The CPP preps used for bead coating were from wt cultures, and thus were identical. In addition, the magnet pulled the beads upward. This should pull the bead farther from the cell surface, making secondary receptor engagement less likely. The possibility remains, however, that pilus differences or secondary receptor engagement may act in concert with membrane tension to generate the higher levels of MAPK activation and gene expression changes seen with infection. Further research is needed to examine this possibility. Nonetheless, we are confident that force plays at least some role in the signaling events identified through this work.

We have begun to assess the biological functions of enhanced gene expression and MAPK activation during gonococcal infection. ERK, JNK, and P38 play a role in determining cell survival during stress and entry into the apoptosis pathway [32]. Moreover, nearly half of the identified retraction-enhanced genes are known to be involved in cell cycle/survival signaling. We show that cells infected with wt bacteria have lower levels of cleaved PARP and cleaved caspase 8 than do uninfected and *pilT*-infected cells. Pilus retraction is therefore predicted to enhance the ability of the cell to withstand apoptosis-inducing signals generated by infection. Indeed, cells infected with wt bacteria withstood STS-induced apoptosis better than uninfected cells and cells infected with *pilT*.

The effect of N. gonorrhoeae infection on cell fate has been a long-standing controversy. The neisserial porin has been reported to protect cells from apoptosis [43] as well as to induce programmed cell death [44]. These conflicting observations are likely a result of differences in experimental systems and bacterial strains. We believe that our results may clarify the issue of N. gonorrhoeae and programmed cell death, through the identification of another bacterial factor (i.e., pilus retraction) involved in such a response.

A number of factors influence the ability of the cell to withstand apoptosis, including the signaling cascades that are activated and the degree and duration of the activation of these cascades [32]. They also include the virulence genes expressed by the infecting bacteria. The bacterial strains used for previous studies on N. gonorrhoeae and apoptosis differed in their piliation state and their ability to invade the host cell. Our results indicate that piliated bacteria, in the absence of pilus retraction, slightly increase the tendency of the infected cell to undergo apoptosis (see Figure 5A). However, these bacteria are still able to moderately protect infected cells from STS-induced apoptosis, indicating that a certain level of cytoprotection is provided by other bacterial factors. In contrast, bacteria that can retract their pili, and thus presumably induce mechanical stress on the host-cell membrane, strongly mediate pro-survival signaling.

The influence of mechanical stress on apoptosis has been studied in some detail. Importantly, such studies indicate that different stress patterns result in different cellular outcomes. Extended, repetitive mechanical force increases the expression of genes encoding cytoprotective heat shock proteins and lowers the number of apoptotic cells in a culture [45]. Suppression of apoptosis requires permanent membrane tension or rhythmic, pulsatile forces [46], which are thought to allow the cell to adapt to new environmental conditions. Retraction events in N. gonorrhoeae generate strong, pulsatile forces every 1–20 s [13]. The nature of the pilus retraction force may therefore be the key to counteracting infection-induced apoptosis. Our data strongly indicate that pilus retraction from a microcolony is capable of stimulating mechanoprotective signals.

**Figure 6:**
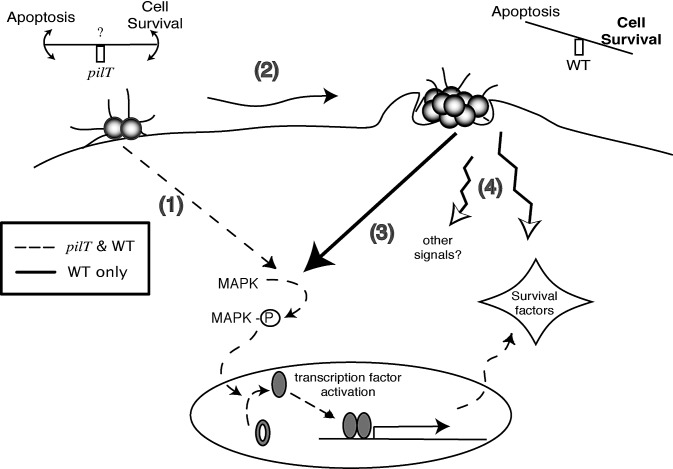
Model of the Role of Pilus Retraction in Promoting a Cytoprotective Environment during Gonococcal Infection of an Epithelial Cell (1) Initial contact between the bacterium and host cell activates low levels of MAPK, and transcription of infection-induced genes. This level of signaling may or may not be able to protect the cell from apoptosis; thus, the host cell “teeters” on the edge of life and death. (2) As the infection proceeds, microcolonies of gonococci are formed, and more pili are locally available to retract. (3) Pilus retraction amplifies MAPK activation, which in turn enhances the transcription of mechanical stress–induced genes. (4) Pilus retraction may also stimulate other pathways that mediate gene expression and survival signaling. Overall signaling events tip the balance in favor of cell survival.

In light of the results presented here and elsewhere, we propose a model to explain how pilus retraction by N. gonorrhoeae influences survival signaling in the infected cell (Figure 6). Initial contact between the bacterium and the epithelial cell activates MAP kinases and alters gene expression at a low level. The cell senses “stress” from the infection, the degree of which varies depending on the metabolic state of the cell and the constellation of virulence factors expressed by the infecting strain. As a result of this stress, the cell is poised to enter the apoptosis pathway. In the absence of pilus retraction and membrane tension, the low levels of activated MAP kinases may or may not be enough to counteract this stress. As the infection proceeds, microcolonies are formed. Pilus retraction from microcolonies is hypothesized to exert stress forces on the membrane, amplifying the levels of activated MAPK, enhancing the transcription of infection-induced genes, and possibly activating other as-yet-unidentified pathways. The end result is the enhanced stimulation of pro-survival pathways and an overriding of pro-apoptotic stress signals. In other words, the fate of the infected cell is decided by the type of signaling networks induced by infection and the extent of activation of these networks. Pilus retraction tips the balance in favor of cell survival.

We have used a tissue culture system to study the interplay between pilus retraction, host cell signaling, and gene expression during the attachment phase of N. gonorrhoeae infection. How these interactions may affect the disease in vivo remains to be clarified. Our results make teleologic sense when the bacterial life cycle and gonococcal disease are taken into consideration. N. gonorrhoeae does not survive on fomites and has no intermediate host. Transmission depends on person-to-person spread. Simple mucosal gonorrhea infections can be mild, and inflammatory responses begin days after exposure [47]. Moreover, a significant number of infected individuals carry gonococci without overt symptoms of disease [47,48]. Indeed, the ability of the bacterium to survive as a species requires a relatively healthy host. Our model for pilus retraction is consistent with these considerations.

## Materials and Methods

### 

#### Reagents

Antibodies to PARP, caspase 8, c-Jun, phospho-c-Jun (Ser63), P44/42 MAPK, phospho-p44/42 MAPK (Thr202/Tyr204), phospho-MAPKAPK2 (Thr334), p38 MAPK, phospho-p38 MAPK (Thr108/Tyr182), SAPK/JNK, and phospho-SAPK/JNK (Thr183/Tyr185) were purchased from Cell Signaling Technology (Beverly, Massachusetts, United States). MAPK inhibitors SB203588, U0126, and SP600125 were purchased from Calbiochem (San Diego, California, United States) and used at a final concentration of 10 μM unless otherwise stated. STS was purchased from Cell Signaling Technology and used at a final concentration of 1 μM to induce apoptosis. Neodymium iron boron (NdFeB) magnets (Eneflux Armtek Magnetics, Bethpage, New York, United States) measured 2 in. in diameter by 1 in. thick and were grade 30 (MGOe).

#### Cell lines, bacterial strains, and infections

T84 human colonic epidermoid cells (American Type Culture Collection, Manassas, Virginia, United States) were maintained in DMEM-F-12 plus 5% heat-inactivated, filter-sterilized fetal bovine serum at 37 °C and 5% CO_2_. For all experiments, cells were seeded into 35-mm dishes and allowed to become confluent before infection. N. gonorrhoeae strains N400 and N400*pilT* [49] were used for all infections and were maintained on GCB agar plus Kellogg's supplements at 37 °C and 5% CO_2_. Piliation and Opa phenotypes were monitored by colony morphology. Only piliated, Opa^−^ bacteria were used. For infection experiments, bacteria were resuspended in GCB liquid medium and added to the epithelial cells at a multiplicity of infection of 50.

#### RNA isolation and microarray analysis

T84 cells were infected with N400 or N400*pilT* or treated with GCB medium alone for 3 h. For RNA isolation, labeling, and microarray hybridization procedures, see Protocol S1. Comparative analysis was performed using MAS 5.0 algorithms to determine fold-change values between uninfected and infected samples from the same experiment, with uninfected samples representing the baseline. Statistical analysis was performed on natural-log transformed data using Cyber-T (http://visitor.ics.uci.edu/genex/cybert/). Subsequent data analysis was performed using Excel (Microsoft, Redmond, Washington, United States) and GeneSpring version 4.0 (Silicon Genetics, Redwood City, California, United States). Genes with a “presence call” *p*-value of less then 0.1 across all chips were eliminated from analysis, as were genes that were given a “no change” call across all samples. A gene was identified as differentially regulated if the fold-change was greater than ±1.5 in at least two out of three experiments. “Enhanced” genes were identified by calculating the ratio of the fold-change for the wt-infected cells to the fold-change for the *pilT*-infected cells (W/P). Gene expression was considered to be enhanced by pilus retraction if the W/P, averaged from at least two out of three individual experiments, was greater than 1.5, and the individual W/P from each experiment was greater than1.25.

#### Real-time RT-PCR analysis

One microgram of total RNA (as isolated above) was reverse-transcribed to generate cDNA, using the iScript cDNA synthesis kit (Bio-Rad, Hercules, California, United States). As a control, parallel samples were run in which reverse transcriptase was omitted from the reaction mixture. Quantitative real-time PCR was performed using an ABI PRISM 7000 Sequence Detection System (Applied Biosystems, Foster City, California, United States). Amplification was carried out using TaqMan master mix (Applied Biosystems), and pre-designed TaqMan probes (Assays on Demand, Applied Biosystems) according to the manufacturer's instructions. Assay numbers are given in Table 1. Reactions were performed in triplicate in a 20-μl volume, with the following cycle parameters: 95 °C/10 min enzyme activation, 95 °C/15 s, 60 °C/1 min for 40 cycles. Data analysis was performed using the comparative Ct method (Applied Biosystems) to determine relative expression levels.

**Table 1:**
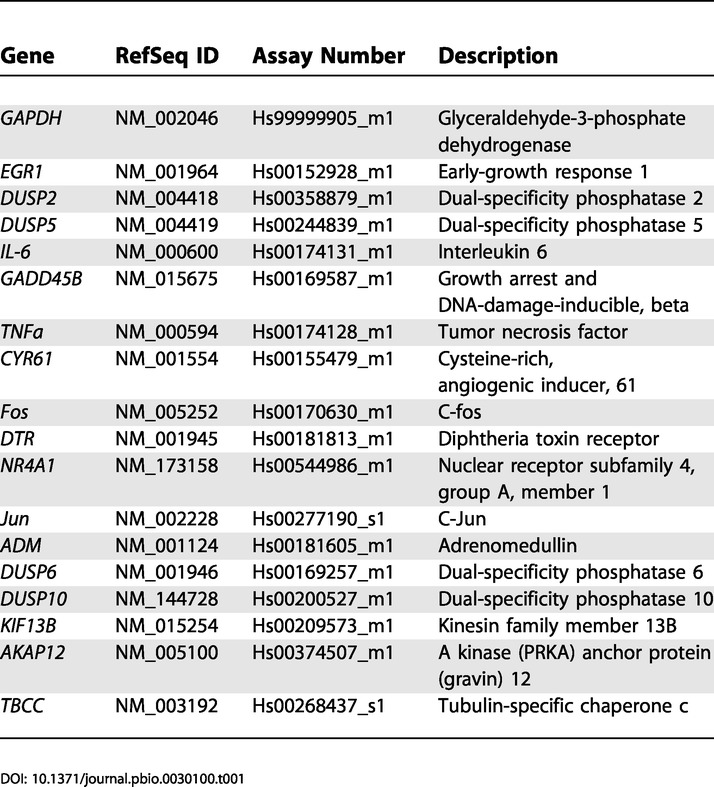
Assays on Demand (TaqMan Probes and Primers) Used for Real-Time Quantitative RT-PCR in This Study

#### Immunoblotting

T84 cells were infected with N400 or N400*pilT* or treated with GCB medium alone for specified times. Following infection, cells were lysed with 150 μl of 1× SDS lysis buffer (62.5 mM Tris-HCl [pH 6.8], 2% w/v SDS, 10% glycerol, 50 mM DTT, 0.1% w/v bromophenol blue), scraped into Eppendorf tubes, vortexed for 15 s, and immediately stored at −20 °C. For PARP and caspase 8 assays, samples were incubated with 150 μl of cell lysis buffer (20 mM Tris [pH 7.5], 150 mM NaCl, 1 mM EDTA, 1 mM EGTA, 1% Triton X-100, 0.5% NP40, 2.5 mM sodium pyrophosphate, 1 mM β-glycerolphosphate, 1 mM Na_3_VO_4_, 1 μg/ml Leupeptin) for 20 min on ice, followed by a 15-s sonication. Samples were boiled for 5 min at 100 °C, then separated by SDS 8% polyacrylamide gels and transferred onto nitrocellulose sheets. Membranes were probed with the specified antibodies following the manufacturer's protocol.

#### CPPs and bead coating

N. gonorrhoeae CPPs were generated from piliated, Opa^−^ gonococci. Bacteria were scraped from overnight cultures (grown on plates) into HBSS and vortexed for 2 min, followed by centrifugation at 14,000*g* for 5 min. Supernatants were removed, quantitated by spectrophotometric analysis, and stored at −80 °C until use. Pili preparations were assayed for the presence of pili via indirect immunofluorescence microscopy and immunoblot, using anti-pilin antibody (data not shown). Bio-Mag Plus carboxy-modified paramagnetic microspheres (Bangs Laboratories, Fishers, Indiana, United States), were activated with 1-ethyl-3-(3-dimethylaminopropyl) carbodiimide, hydrochloride (EDAC), and incubated with piliated N. gonorrhoeae CPPs or BSA as per the manufacturer's instructions. Bead coating was confirmed by immunoblotting using antibodies to BSA (ICN Biomedicals, Irvine, California, United States) and pilin (antibody SM1; data not shown).

#### Immunofluorescence microscopy

T84 cells were grown on coverslips to 50% confluency and incubated with either BSA-coated or CMP-coated magnetic beads for 15 min. Unbound beads were washed off and the magnet placed at a distance of 10 mm from the cell surface for 1 h. The medium was then aspirated, and the cells fixed for 15 min at room temperature in 4% paraformaldehyde. Cells were blocked and permeabilized in isotonic PBS containing BSA (3%, w/v) and saponin (0.02% w/v) for 1 h at room temperature, followed by staining with Alexa-Fluor 594 phalloidin (Molecular Probes, Eugene, Oregon, United States) at 1:1,000 for 30 min. Samples were rinsed extensively in PBS before mounting in Fluoromount-G (Fisher Scientific, Hampton, New Hampshire, United States). Images were obtained with a Deltavision Restoration Microscope (Applied Precision, Issaquah, Washington, United States) fitted with a Nikon (Tokyo, Japan) 60× oil-immersion objective and processed at a Silicon Graphics (Mountain View, California, United States) workstation with accompanying API software. The images were subsequently exported to Adobe Photoshop (version 7.0) and Adobe Illustrator (version 11.0) (Adobe Systems, San Jose, California, United States) for manuscript preparation.

#### Calculation of magnetic force

To quantify the amount of force that the magnet exerts per magnetic bead, the change-in-mass method [31] was used. Briefly, the mass of a known number of dry beads (0.12 g) was measured on an electronic balance in the presence and absence of the magnet. Given the mean bead diameter of 1.5 μm and the bead density of 2.5 × 10^3^ kg/m^3^ (Bangs Laboratories), the number of beads in this sample was calculated to be 1.2 × 10^10^. The change in mass of the beads in the presence of the magnet was entered into the equation—force = Δmass × acceleration (with acceleration being equal to gravity, or 9.81 m/s^2^)—to give a value for the force. Change-in-mass measurements were taken at varying distances from the magnet to determine force as a function of distance (see Figure 4B).

#### Magnetic force experiments

T84 cells were grown to confluency in 35-mm culture dishes. Before assay, the cells were incubated with prewarmed, serum-free medium for 2 h. Cells were then incubated for 30 min with medium alone, or with CPP- or BSA-coated beads diluted in the same medium. Cells were then washed with fresh, serum-free medium to remove unbound beads. Magnets were placed at a distance of 10 mm from the bottom of the tissue culture dish, and the dishes were incubated for the specified time at 37 °C, 5% CO_2_. The samples were then processed for RNA isolation or SDS-PAGE, as described above. Control samples were treated in parallel but were not exposed to the magnet.

#### Statistics

Statistical analysis was performed using standard *t*-test analysis with SPSS version 11.0 (SPSS, Chicago, Illinois, United States) unless otherwise stated.

## Supporting Information

Protocol S1 MIAME Checklist (42 KB DOC).

Table S1 Supplementary References (409 KB DOC).

### Accession Numbers

The GenBank (http://www.ncbi.nlm.nih.gov/Genbank/) accession numbers for the genes and gene products discussed in this paper are *ADM* (D14874), *cyr61* (Y11307), DTR (M60278), *DUSP5* (U15932), EGR1 (X52541), PilF (U32588), and PilT (S72391).

## Abbreviations

BSA: bovine serum albumin
CPP: crude pili preparation
MAPK: mitogen-activated protein kinase
PARP: poly(ADP-ribose) polymerase
STS: staurosporine
Tfp: type IV pili
W/P: wild-type to *pilT* fold-change expression ratio
wt: wild-type
