# Supplementary material for: The N. gonorrhoeae Type IV Pilus Stimulates Mechanosensitive Pathways and Cytoprotection through a pilT-Dependent Mechanism
Source: PLoS Biol. 2005 Mar 22;3(4):e100. doi: 10.1371/journal.pbio.0030100 (PMC1065265; doi:10.1371/journal.pbio.0030100)
Supplement: Supplementary file 1 [file pbio.0030100.sd001.doc]

## BBC MIAME Checklist version 1.0

##  Affymetrix Experiments

**EXPERIMENT DESIGN**

Authors / Submitters

Magdalene So

Heather L. Howie

Department of Molecular Microbiology and Immunology, Oregon Health & Science University, Portland, Oregon.

Type of experiment: for example, is it a comparison of normal vs. diseased tissue, a time course, or is it designed to study the effects of a gene knock-out?

Compared the gene expression profiles of uninfected T84 human epithelial cells to cells infected with wild-type *Neisseria gonorrhoeae*, or an isogenic mutant (*pilT*) that is unable to undergo pilus retraction, to test the hypothesis that pilus retraction alters the host cell transcriptome.

Experimental factors: the parameters or conditions tested, such as time, dose, or genetic variation.

3 experimental conditions:

Uninfected

Wild-type infected (3hr infection with strain N400)

*pilT*-infected (3hr infection with strain N400*pilT*)

The number of hybridizations performed in the experiment.

9 total hybridizations

The type of reference used for the hybridizations, if any.

No references used

Hybridization design: if applicable, a description of the comparisons made in each hybridization, whether to a standard reference sample, or between experimental samples. An accompanying diagram or table may be useful.

Comparisons between uninfected and wild-type infected; comparisons between uninfected and *pilT* infected: comparisons between wild-type infected and *pilT* infected

Quality control steps taken.

Isolated total RNA was analyzed on an Agilent Bioanalyzer 2100 to verify that starting material was of good quality. Standard Affymetrix control steps were then taken. Labeled RNA was hybridized to a Test Array containing probe sets for all Affymetrix Expression Array controls. Background noise, Avg Diff, and ratio of Avg Diff values for probe sets representing the 5’ and 3’ ends of actin and GAPDH transcripts were used to determine target quality. Targets that did not meet the empirically determined cut-off values within the project were re-made. Quality tested samples were then hybridized to the HG_U95Av2 array.

Number of replicates (Biological or Technical)

3 biological replicates for each condition tested

URL of any supplemental websites or database accession numbers.

Data will be hosted on the Oregon Health & Science University Biostatistics and Bioinformatics Shared Resource web page ([www.ohsu.edu/cbcibm/bbsr](http://www.ohsu.edu/cbcibm/bbsr)) following publication.

**SAMPLES USED, EXTRACT PREPARATION AND LABELING**

- **The origin of the biological sample (for instance, name of the organism, the provider of the sample) and its characteristics: for example, gender, age, developmental stage, strain, or disease state.**

Organism: Homo Sapien

Cell line: T84 (derived from human colorectal cancer)

Passage: 4-9

Bacterial strains: N400 and N400*pilT*

Bacterial strain origin: Michael Koomey

Passage: 4-6

Manipulation of biological samples and protocols used: for example, growth conditions, treatments, separation techniques.

Fresh DMEM-F12 media (+ 5% FBS) was added to confluent T84 cells seeded onto wells of a 6-well plate. N400 or N400*pilT* suspended in GCB media were added to the cell monolayers at an MOI (multiplicity of infection) of 50. Uninfected cells were treated with an equivalent volume of GCB media. Plates were incubated at 37ºC/5% CO_2_ for 3 hours. Following incubation, media was aspirated and replaced with buffer RLT (+ beta-mercaptoethanol). Cells were scraped off of plates, and transferred to qiashredder columns (Qiagen Inc.) to homogenize each sample, followed by storage at -80ºC.

Protocol for preparing the hybridization extract: for example, the RNA or DNA extraction and purification protocol.

Total RNA was isolated using the Qiagen RNeasy kit (Qiagen Inc.).

Labeling protocol(s).

Standard Affymetrix Protocols (Affymetrix GeneChip Expression Analysis Technical Manual, rev.3. 2001)

External controls (spikes).

Standard Affymetrix external spikes added to hybridization mixture: BioB, BioC, BioD and CreX at 1.5pm, 5.0pm, 25pm and 100pm respectively,

HYBRIDIZATION PROCEDURES AND PARAMETERS

The protocol and conditions used during hybridization, blocking and washing.

Standard Affymetrix Protocols (Affymetrix GeneChip Expression Analysis Technical Manual, rev.3. 2001)

MEASUREMENT DATA AND SPECIFICATIONS

Quantitations based on the images

Original Affymetrix .dat proprietory output files

Type of scanning hardware and software used: this information is appropriate for a materials and methods section.

Software – Affymetrix Microarray Suite 5.0

Scanning Hardware – HP GeneArray Scanner

Type of image analysis software used: specifications should be stated in the materials and methods.

Affymetrix Microarray Suite 5.0 was used for image analysis and feature extraction

A description of the measurements produced by the image-analysis software and a description of which measurements were used in the analysis.

Probe level measurements produced by Affymetrix Microarray Suite 5.0. Analysis performed at level of raw signal. (See Affymetrix Microarray Suite 5.0 reference on web site for description).

The complete output of the image analysis *before* data selection and transformation (spot quantitation matrices).

Original Affymetrix output files

Data selection and transformation procedures.

Genes displaying a “detection p-value” less than 0.1 in 8 or more arrays (out of a total of 9 arrays) were removed from the analysis. Regulated genes were identified based on the following criteria:

1) A 1.5 fold difference in expression in wild-type and/or *pilT* infected cells as compared to uninfected cells in at least 2 out of 3 replicates.
2) An average W/P ratio (wild-type fold-change divided by *pilT* fold-change) of ≥ 1.5 or ≤ .67 from 2 out of 3 replicates
3) Both values used to calculate average W/P ratios must be ≥1.25 or ≤ 0.8

A gene was considered for further analysis only if condition (1) was met. A gene was considered to be regulated by pilus retraction if both conditions (2) and (3) were met.

For statistical analysis, data were ln transformed, and analyzed using CyberT (<http://visitor.ics.uci.edu/genex/cybert>) to generate p-values from regularized t-tests using a Bayesian estimate of the variance among gene measurements within an experiment. These p-values are used only as supporting data for the genes identified by the three criteria listed above.

Final gene expression data table(s) used by the authors to make their conclusions *after* data selection and transformation (gene expression data matrices).

See worksheet Final.xls for results

# ARRAY DESIGN

- **Platform Type**

Synthesized Oligonucleotide Array

- **Surface and Coating Specifications**

Glass

- **Availability of the Array (name/make)**

Affymetrix Human Genome HG_U95Av2

- **For each feature (spot) on the array, its location on the array and the ID of its respective reporter (molecule present on each spot) should be given**.

See www.affymetrix.com

- **For each reporter, its type (e.g., cDNA or oligonucleotide) should be given, along with information that characterizes the reporter molecule unambiguously, in the form of appropriate database reference(s) and sequence (if available).**

See www.affymetrix.com
