# Supplementary material for: The N. gonorrhoeae Type IV Pilus Stimulates Mechanosensitive Pathways and Cytoprotection through a pilT-Dependent Mechanism
Source: PLoS Biol. 2005 Mar 22;3(4):e100. doi: 10.1371/journal.pbio.0030100 (PMC1065265; doi:10.1371/journal.pbio.0030100)
Supplement: Supplementary file 2 [file pbio.0030100.st001.doc]

| **Gene** | **Affy ID** | **GenBank** | **Function** | **Induction** | **References (in addition to Locus Link descriptions)** |
| --- | --- | --- | --- | --- | --- |
| NR4A1 | 279_at | L13740 | TC,CS,SI | MS,ES,MAPK | (Bandoh et al. 1997; Honkaniemi et al. 2000; Li et al. 2000; Pekarsky  et al. 2001; Song et al. 2002; Suzuki et al. 2003) |
| NR4A1 | 280_g_at | L13740 | TC,CS,SI | MS,ES,MAPK | (Bandoh et al. 1997; Honkaniemi et al. 2000; Li et al. 2000; Pekarsky  et al. 2001; Song et al. 2002; Suzuki et al. 2003) |
| Fos | 2094_s_at | K00650 | TC,CS | MS,ES,MAPK | (Li et al. 2002) |
| IL-8 | 1369_s_at | M28130 | SI | MS,ES,MAPK | (Hoffmann et al. 2002; Liang et al. 2002a; Liang et al. 2002b; Oudin and  Pugin 2002; Yamamoto et al. 2002; Akhtar et al. 2003; Chen et al. 2003) |
| DUSP1 | 1005_at | X68277 | KP,CS,SI | MS,ES,MAPK | (Li et al. 1999; Denkert et al. 2002; Theodosiou and Ashworth 2002) |
| IL-8 | 35372_r_at | M17017 | SI | MS,ES,MAPK | (Hoffmann et al. 2002; Liang et al. 2002a; Liang et al. 2002b; Oudin and  Pugin 2002; Yamamoto et al. 2002; Akhtar et al. 2003; Chen et al. 2003) |
| IL-6 | 38299_at | X04430 | SI | MS,ES,MAPK | (Kishikawa et al. 2002; Meduri 2002; Naka et al. 2002; Kobayashi et al.  2003; Mann 2003) |
| DUSP5 | 529_at | U15932 | KP | MS,ES,MAPK | (Ueda et al. 2003) |
| NR4A2 | 547_s_at | S77154 | TC | MS,ES,MAPK | (Bandoh et al. 1997; Maira et al. 1999; Honkaniemi et al. 2000; Chai and  Tarnawski 2002; Park et al. 2003) |
| EGR2 | 37863_at | J04076 | TC,CS | CC,MAPK | (Chavrier et al. 1988; Hirano et al. 2002; Unoki and Nakamura 2003;  Zeng et al. 2003) |
| DTR | 38037_at | M60278 | CS,SI | MS,ES,MAPK | (Park et al. 1999; Kuhn et al. 2002; Tschumperlin et al. 2002; Wallasch  et al. 2002; Ingram et al. 2003; Xia et al. 2003) |
| Gro1 | 408_at | X54489 | CS,SI | MS,ES | (Hagiwara et al. 1998; Dunican et al. 2000; Thorpe et al. 2001; Dhawan  and Richmond 2002; Wang et al. 2003) |
| Gro2 | 37187_at | M36820 | CS,SI | MS,ES | (Joshi-Barve et al. 1993; Hess et al. 2001; Quinn et al. 2002; Tarzami  et al. 2002) |
| TNFa | 1852_at | X02910 | CS,SI | MS,ES,MAPK | (Long et al. 2001; Gupta 2002; Hehlgans and Mannel 2002; Palmieri et al.  2002; Lamb et al. 2003) |
| SOCS3 | 40968_at | AB004904 | SI | MS,ES,MAPK | (Bode et al. 2001; Krebs and Hilton 2001; Yasukawa et al. 2001; Mysorekar  et al. 2002) |
| JunB | 2049_s_at | M29039 | TC,CS | MS,ES,MAPK | (Balmanno and Cook 1999; Frazier-Jessen et al. 2002; Granet et al. 2002;  Shaulian and Karin 2002) |
| Id1 | 36619_r_at | S78825 | TC,CS | U | (Miyazono and Miyazawa 2002; Kang et al. 2003; Sikder et al. 2003) |
| TCF8 | 33439_at | D15050 | TC | U | (Grooteclaes and Frisch 2000; Postigo et al. 2003) |
| Gro3 | 34022_at | M36821 | CA,SI | MS,ES | (Haskill et al. 1990; Yang et al. 1997; Hagiwara et al. 1998; Curnock et al. 2002) |
| H2BFR | 35562_at | AI076718 | TC | CC | (LaBella et al. 1988) |
| H2AF0 | 32609_at | AI885852 | TC | CC | (Oswald et al. 1996; Augusto et al. 2003) |
| BLIMP1 | 31779_s_at | AF084199 | SI | ES | (Schebesta et al. 2002; Gyory et al. 2003) |
| ATF3 | 287_at | L19871 | TC,CS,SI | MS,ES,MAPK | (Liang et al. 1996; Hai et al. 1999; Kawauchi et al. 2002; Sepulveda et al.  2002; Kool et al. 2003; Nakagomi et al. 2003) |
| KRT16 | 601_s_at | M28439 | CS,CA | MS,CC | (Jiang et al. 1993; Paramio et al. 1999; McClowry et al. 2002) |
| CSF2 | 1400_at | M13207 | CS,SI | MS,ES,MAPK | (Kosaki et al. 1998; Reibman et al. 2002; Quentmeier et al. 2003) |
| NR4A2 | 37623_at | X75918 | TC | MS,ES,MAPK | (Bandoh et al. 1997; Maira et al. 1999; Honkaniemi et al. 2000; Chai and  Tarnawski 2002; Park et al. 2003) |
| CYR61 | 38772_at | Y11307 | CS,CA,SI | MS,ES,MAPK | (Lau and Lam 1999; Tamura et al. 2001; Leu et al. 2002; Brigstock 2003;  Han et al. 2003; Kunz et al. 2003) |
| H2AF0 | 286_at | L19779 | TC | CC | (Oswald et al. 1996; Augusto et al. 2003) |
| CSF2 | 1401_g_at | M13207 | CS,SI | MS,ES,MAPK | (Kosaki et al. 1998; Reibman et al. 2002; Quentmeier et al. 2003) |
| DUSP2 | 1292_at | L11329 | KP,CS | MS,ES,MAPK | (Rohan et al. 1993; Yin et al. 2003) |
| EGR1 | 789_at | X52541 | TC | MS,ES,MAPK | (Schwachtgen et al. 1998; O'Donovan et al. 1999; Silverman et al. 1999; Stula  et al. 2000) |
| H2AFA | 35127_at | AI039144 | TC | CC | (Mannironi et al. 1994; Bosch and Suau 1995) |
| OVOL1 | 34047_at | AF016045 | TC | U | - - - - |
| uPA | 37310_at | X02419 | CS,CA,SI | MS,ES,MAPK | (Wang et al. 1995; Irigoyen et al. 1997; Aguirre Ghiso et al. 1999; Blasi 1999;  Cirillo et al. 1999; Redmond et al. 1999; Miura et al. 2000; Essig et al. 2001;  Stefansson and Lawrence 2003; Tarui et al. 2003; Watanabe et al. 2003) |
| Gadd45B | 39822_s_at | AF078077 | CS,SI | ES | (De Smaele et al. 2001; Kyriakis 2001; Jin et al. 2002; Liebermann and Hoffman  2002, 2003) |
| Gadd34 | 37028_at | U83981 | CS,SI | ES,MAPK | (Oh-Hashi et al. 2001; Liebermann and Hoffman 2002; Sarkar et al. 2002; Hung  et al. 2003) |
| RhoB | 1826_at | M12174 | CS,CA,SI | ES | (Jahner and Hunter 1991; Zalcman et al. 1995; Gampel et al. 1999; Fritz and  Kaina 2001a, 2001b; Prendergast 2001; Ridley 2001) |
|  | 37538_at | AL049354 | U | U | - - - - |
| HSP70-2 | 31692_at | M59830 | CS,SI | MS,ES | (Volloch et al. 2000; Park et al. 2001; Pirkkala et al. 2001; Gabai et al. 2002;  Gabai and Sherman 2002; Galvin et al. 2002; Nollen and Morimoto 2002;  Liu et al. 2003) |
| H1F2 | 37018_at | AI189287 | TC | CC | (Marzluff et al. 2002) |
| Id1 | 36618_g_at | X77956 | TC,CS | U | (Miyazono and Miyazawa 2002; Kang et al. 2003; Sikder et al. 2003) |
| KIP2 | 38673_s_at | D64137 | KP,CS,SI | MS,ES | (Billotte et al. 2001; Hsu et al. 2002; Petermann et al. 2002; Chang et al. 2003) |
| HBP17 | 38489_at | M60047 | CS | MAPK | (Harris et al. 2000; Chen et al. 2001; Tassi et al. 2001) |
| Rho6 | 37785_at | U69563 | CA | U | (Nobes et al. 1998; Chardin 1999; Katoh et al. 2002; Oinuma et al. 2003;  Wennerberg et al. 2003) |
| MIP1A | 36103_at | D90144 | SI | ES | (Shi et al. 1996; Takahashi et al. 2002; Li et al. 2003) |
| HSPF1 | 752_s_at | D85429 | SI | MS,ES | (Ohtsuka and Hata 2000; Sironen et al. 2002) |
| MEKK1 | 33009_at | AF042838 | KP,CS | U | (Schlesinger et al. 1998) |
| KIP2 | 1787_at | U22398 | KP,CS,SI | MS,ES | (Billotte et al. 2001; Hsu et al. 2002; Petermann et al. 2002; Chang et al. 2003) |
| H3FB | 34964_at | N35832 | TC | CC | (Frank et al. 2003) |
|  | 38207_at | AW006742 | U | U | - - - - |
| E2F2 | 37043_at | AL021154 | TC,CS | CC | (Leone et al. 2001; de Bruin et al. 2003; Zhu et al. 2003) |
| CYP1A1 | 1024_at | X02612 | SI | ES | (Morgan et al. 1998; Barouki and Morel 2001) |
| FDZF2 | 38872_at | U95044 | TC | U | - - - - |
|  | 36070_at | AL049389 | U | U | - - - - |
| ZNF253 | 35573_r_at | AF038951 | TC | U | (Han et al. 1999) |
| PLXNC1 | 32193_at | AF030339 | CA | U | (Comeau et al. 1998; Pasterkamp et al. 2003) |
| ALDH1A3 | 36686_at | U07919 | SI | U | (Okamura et al. 1999; Pappas et al. 2001) |
| CYP1A1 | 1025_g_at | X02612 | SI | ES | (Morgan et al. 1998; Barouki and Morel 2001) |
| CYP1A1 | 36767_at | K03191 | SI | ES | (Morgan et al. 1998; Barouki and Morel 2001) |
|  | 40143_at | D50930 | U | U | - - - - |
| SOCS5 | 32669_at | AB014571 | SI | ES | (Yasukawa et al. 2000) |
|  | 34283_at | AL050125 | U | U | - - - - |
|  | 40848_g_at | AB018293 | U | U | - - - - |
| CBX6 | 39560_at | H10776 | TC | U | (Jones et al. 2001) |
| SIM2 | 39608_at | U80456 | TC | U | - - - - |
|  | 41807_at | AL040137 | U | U | - - - - |
|  | 38505_at | AL050151 | U | U | - - - - |
| GRLF1 | 34724_at | AI670100 | CA | U | (Brouns et al. 2000; Zangerl et al. 2002) |
| ZNF211 | 38142_at | U38904 | TC,CS | U | (Becker et al. 1997) |

Functions: TC (transcription factor), KP (kinase/phosphatase), CS (cell cycle/survival), CA (cytoskeleton/adhesion), SI (stress/immune response) and U (unknown)

Induction mechanisms: MS (mechanical stress), ES (environmental stress), CC (cell cycle), MAPK (mitogen activated protein kinase) and U (unknown)

Aguirre Ghiso JA, Alonso DF, Farias EF, Gomez DE, de Kier Joffe EB (1999) Deregulation of the signaling pathways controlling urokinase production. Its relationship with the invasive phenotype. Eur J Biochem 263(2): 295-304.

Akhtar M, Watson JL, Nazli A, McKay DM (2003) Bacterial DNA evokes epithelial IL-8 production by a MAPK-dependent, NF-kappaB-independent pathway. Faseb J 17(10): 1319-1321.

Augusto LA, Decottignies P, Synguelakis M, Nicaise M, Le Marechal P, et al. (2003) Histones: a novel class of lipopolysaccharide-binding molecules. Biochemistry 42(13): 3929-3938.

Balmanno K, Cook SJ (1999) Sustained MAP kinase activation is required for the expression of cyclin D1, p21Cip1 and a subset of AP-1 proteins in CCL39 cells. Oncogene 18(20): 3085-3097.

Bandoh S, Tsukada T, Maruyama K, Ohkura N, Yamaguchi K (1997) Mechanical agitation induces gene expression of NOR-1 and its closely related orphan nuclear receptors in leukemic cell lines. Leukemia 11(9): 1453-1458.

Barouki R, Morel Y (2001) Repression of cytochrome P450 1A1 gene expression by oxidative stress: mechanisms and biological implications. Biochem Pharmacol 61(5): 511-516.

Becker KG, Canning RD, Nagle JW, Dehejia AM, Polymeropoulos MH, et al. (1997) Molecular cloning and mapping of a novel developmentally regulated human C2H2-type zinc finger. Mamm Genome 8(4): 287-289.

Billotte WG, Dumas K, Hofmann MC (2001) Transcriptional pathways induced by fluid shear stress in mouse preosteoblast cells. Biomed Sci Instrum 37: 1-6.

Blasi F (1999) The urokinase receptor. A cell surface, regulated chemokine. Apmis 107(1): 96-101.

Bode JG, Ludwig S, Freitas CA, Schaper F, Ruhl M, et al. (2001) The MKK6/p38 mitogen-activated protein kinase pathway is capable of inducing SOCS3 gene expression and inhibits IL-6-induced transcription. Biol Chem 382(10): 1447-1453.

Bosch A, Suau P (1995) Changes in core histone variant composition in differentiating neurons: the roles of differential turnover and synthesis rates. Eur J Cell Biol 68(3): 220-225.

Brigstock DR (2003) The CCN family: a new stimulus package. J Endocrinol 178(2): 169-175.

Brouns MR, Matheson SF, Hu KQ, Delalle I, Caviness VS, et al. (2000) The adhesion signaling molecule p190 RhoGAP is required for morphogenetic processes in neural development. Development 127(22): 4891-4903.

Chai J, Tarnawski AS (2002) Serum response factor: discovery, biochemistry, biological roles and implications for tissue injury healing. J Physiol Pharmacol 53(2): 147-157.

Chang TS, Kim MJ, Ryoo K, Park J, Eom SJ, et al. (2003) p57KIP2 modulates stress-activated signaling by inhibiting c-Jun NH2-terminal kinase/stress-activated protein Kinase. J Biol Chem 278(48): 48092-48098. Epub 42003 Sep 48098.

Chardin P (1999) Rnd proteins: a new family of Rho-related proteins that interfere with the assembly of filamentous actin structures and cell adhesion. Prog Mol Subcell Biol 22: 39-50.

Chavrier P, Zerial M, Lemaire P, Almendral J, Bravo R, et al. (1988) A gene encoding a protein with zinc fingers is activated during G0/G1 transition in cultured cells. Embo J 7(1): 29-35.

Chen H, Wu L, Liu X, Chen Y, Wang B (2003) Effects of laminar shear stress on IL-8 mRNA expression in endothelial cells. Biorheology 40(1-3): 53-58.

Chen JH, Wang XC, Sato JD (2001) Bifunctional effects of heparin-binding protein HBp17 on DNA synthesis in cells. Cell Biol Int 25(6): 567-570.

Cirillo G, Casalino L, Vallone D, Caracciolo A, De Cesare D, et al. (1999) Role of distinct mitogen-activated protein kinase pathways and cooperation between Ets-2, ATF-2, and Jun family members in human urokinase-type plasminogen activator gene induction by interleukin-1 and tetradecanoyl phorbol acetate. Mol Cell Biol 19(9): 6240-6252.

Comeau MR, Johnson R, DuBose RF, Petersen M, Gearing P, et al. (1998) A poxvirus-encoded semaphorin induces cytokine production from monocytes and binds to a novel cellular semaphorin receptor, VESPR. Immunity 8(4): 473-482.

Curnock AP, Logan MK, Ward SG (2002) Chemokine signalling: pivoting around multiple phosphoinositide 3-kinases. Immunology 105(2): 125-136.

de Bruin A, Maiti B, Jakoi L, Timmers C, Buerki R, et al. (2003) Identification and characterization of E2F7, a novel mammalian E2F family member capable of blocking cellular proliferation. J Biol Chem 278(43): 42041-42049. Epub 42003 Jul 42031.

De Smaele E, Zazzeroni F, Papa S, Nguyen DU, Jin R, et al. (2001) Induction of gadd45beta by NF-kappaB downregulates pro-apoptotic JNK signalling. Nature 414(6861): 308-313.

Denkert C, Schmitt WD, Berger S, Reles A, Pest S, et al. (2002) Expression of mitogen-activated protein kinase phosphatase-1 (MKP-1) in primary human ovarian carcinoma. Int J Cancer 102(5): 507-513.

Dhawan P, Richmond A (2002) Role of CXCL1 in tumorigenesis of melanoma. J Leukoc Biol 72(1): 9-18.

Dunican AL, Leuenroth SJ, Ayala A, Simms HH (2000) CXC chemokine suppression of polymorphonuclear leukocytes apoptosis and preservation of function is oxidative stress independent. Shock 13(3): 244-250.

Essig M, Terzi F, Burtin M, Friedlander G (2001) Mechanical strains induced by tubular flow affect the phenotype of proximal tubular cells. Am J Physiol Renal Physiol 281(4): F751-762.

Frank D, Doenecke D, Albig W (2003) Differential expression of human replacement and cell cycle dependent H3 histone genes. Gene 312: 135-143.

Frazier-Jessen MR, Thompson CD, Brown R, Rawat R, Nordan RP, et al. (2002) NF-kappaB elements contribute to junB inducibility by lipopolysaccharide in the murine macrophage cell line RAW264.7. FEBS Lett 513(2-3): 203-207.

Fritz G, Kaina B (2001a) Ras-related GTPase Rhob represses NF-kappaB signaling. J Biol Chem 276(5): 3115-3122. Epub 2000 Nov 3112.

Fritz G, Kaina B (2001b) Transcriptional activation of the small GTPase gene rhoB by genotoxic stress is regulated via a CCAAT element. Nucleic Acids Res 29(3): 792-798.

Gabai VL, Sherman MY (2002) Invited review: Interplay between molecular chaperones and signaling pathways in survival of heat shock. J Appl Physiol 92(4): 1743-1748.

Gabai VL, Mabuchi K, Mosser DD, Sherman MY (2002) Hsp72 and stress kinase c-jun N-terminal kinase regulate the bid-dependent pathway in tumor necrosis factor-induced apoptosis. Mol Cell Biol 22(10): 3415-3424.

Galvin DJ, Watson RW, Gillespie JI, Brady H, Fitzpatrick JM (2002) Mechanical stretch regulates cell survival in human bladder smooth muscle cells in vitro. Am J Physiol Renal Physiol 283(6): F1192-1199. Epub 2002 Jul 1130.

Gampel A, Parker PJ, Mellor H (1999) Regulation of epidermal growth factor receptor traffic by the small GTPase rhoB. Curr Biol 9(17): 955-958.

Granet C, Vico AG, Alexandre C, Lafage-Proust MH (2002) MAP and src kinases control the induction of AP-1 members in response to changes in mechanical environment in osteoblastic cells. Cell Signal 14(8): 679-688.

Grooteclaes ML, Frisch SM (2000) Evidence for a function of CtBP in epithelial gene regulation and anoikis. Oncogene 19(33): 3823-3828.

Gupta S (2002) A decision between life and death during TNF-alpha-induced signaling. J Clin Immunol 22(4): 185-194.

Gyory I, Fejer G, Ghosh N, Seto E, Wright KL (2003) Identification of a functionally impaired positive regulatory domain I binding factor 1 transcription repressor in myeloma cell lines. J Immunol 170(6): 3125-3133.

Hagiwara H, Mitsumata M, Yamane T, Jin X, Yoshida Y (1998) Laminar shear stress-induced GRO mRNA and protein expression in endothelial cells. Circulation 98(23): 2584-2590.

Hai T, Wolfgang CD, Marsee DK, Allen AE, Sivaprasad U (1999) ATF3 and stress responses. Gene Expr 7(4-6): 321-335.

Han JS, Macarak E, Rosenbloom J, Chung KC, Chaqour B (2003) Regulation of Cyr61/CCN1 gene expression through RhoA GTPase and p38MAPK signaling pathways. Eur J Biochem 270(16): 3408-3421.

Han ZG, Zhang QH, Ye M, Kan LX, Gu BW, et al. (1999) Molecular cloning of six novel Kruppel-like zinc finger genes from hematopoietic cells and identification of a novel transregulatory domain KRNB. J Biol Chem 274(50): 35741-35748.

Harris VK, Coticchia CM, Kagan BL, Ahmad S, Wellstein A, et al. (2000) Induction of the angiogenic modulator fibroblast growth factor-binding protein by epidermal growth factor is mediated through both MEK/ERK and p38 signal transduction pathways. J Biol Chem 275(15): 10802-10811.

Haskill S, Peace A, Morris J, Sporn SA, Anisowicz A, et al. (1990) Identification of three related human GRO genes encoding cytokine functions. Proc Natl Acad Sci U S A 87(19): 7732-7736.

Hehlgans T, Mannel DN (2002) The TNF-TNF receptor system. Biol Chem 383(10): 1581-1585.

Hess S, Rheinheimer C, Tidow F, Bartling G, Kaps C, et al. (2001) The reprogrammed host: Chlamydia trachomatis-induced up-regulation of glycoprotein 130 cytokines, transcription factors, and antiapoptotic genes. Arthritis Rheum 44(10): 2392-2401.

Hirano S, Anuradha CD, Kanno S (2002) krox-20/egr-2 is up-regulated following non-specific and homophilic adhesion in rat macrophages. Immunology 107(1): 86-92.

Hoffmann E, Dittrich-Breiholz O, Holtmann H, Kracht M (2002) Multiple control of interleukin-8 gene expression. J Leukoc Biol 72(5): 847-855.

Honkaniemi J, Zhang JS, Longo FM, Sharp FR (2000) Stress induces zinc finger immediate early genes in the rat adrenal gland. Brain Res 877(2): 203-208.

Hsu S, Yu FS, Lewis J, Singh B, Borke J, et al. (2002) Induction of p57 is required for cell survival when exposed to green tea polyphenols. Anticancer Res 22(6C): 4115-4120.

Hung WJ, Roberson RS, Taft J, Wu DY (2003) Human BAG-1 proteins bind to the cellular stress response protein GADD34 and interfere with GADD34 functions. Mol Cell Biol 23(10): 3477-3486.

Ingram JL, Rice AB, Santos J, Van Houten B, Bonner JC (2003) Vanadium-induced HB-EGF expression in human lung fibroblasts is oxidant dependent and requires MAP kinases. Am J Physiol Lung Cell Mol Physiol 284(5): L774-782. Epub 2003 Jan 2010.

Irigoyen JP, Besser D, Nagamine Y (1997) Cytoskeleton reorganization induces the urokinase-type plasminogen activator gene via the Ras/extracellular signal-regulated kinase (ERK) signaling pathway. J Biol Chem 272(3): 1904-1909.

Jahner D, Hunter T (1991) The ras-related gene rhoB is an immediate-early gene inducible by v-Fps, epidermal growth factor, and platelet-derived growth factor in rat fibroblasts. Mol Cell Biol 11(7): 3682-3690.

Jiang CK, Magnaldo T, Ohtsuki M, Freedberg IM, Bernerd F, et al. (1993) Epidermal growth factor and transforming growth factor alpha specifically induce the activation- and hyperproliferation-associated keratins 6 and 16. Proc Natl Acad Sci U S A 90(14): 6786-6790.

Jin R, De Smaele E, Zazzeroni F, Nguyen DU, Papa S, et al. (2002) Regulation of the gadd45beta promoter by NF-kappaB. DNA Cell Biol 21(7): 491-503.

Jones DO, Mattei MG, Horsley D, Cowell IG, Singh PB (2001) The gene and pseudogenes of Cbx3/mHP1 gamma. DNA Seq 12(3): 147-160.

Joshi-Barve SS, Rangnekar VV, Sells SF, Rangnekar VM (1993) Interleukin-1-inducible expression of gro-beta via NF-kappa B activation is dependent upon tyrosine kinase signaling. J Biol Chem 268(24): 18018-18029.

Kang Y, Chen CR, Massague J (2003) A self-enabling TGFbeta response coupled to stress signaling: Smad engages stress response factor ATF3 for Id1 repression in epithelial cells. Mol Cell 11(4): 915-926.

Katoh H, Harada A, Mori K, Negishi M (2002) Socius is a novel Rnd GTPase-interacting protein involved in disassembly of actin stress fibers. Mol Cell Biol 22(9): 2952-2964.

Kawauchi J, Zhang C, Nobori K, Hashimoto Y, Adachi MT, et al. (2002) Transcriptional repressor activating transcription factor 3 protects human umbilical vein endothelial cells from tumor necrosis factor-alpha-induced apoptosis through down-regulation of p53 transcription. J Biol Chem 277(41): 39025-39034. Epub 32002 Aug 39022.

Kishikawa H, Miura S, Yoshida H, Hirokawa M, Nakamizo H, et al. (2002) Transmural pressure induces IL-6 secretion by intestinal epithelial cells. Clin Exp Immunol 129(1): 86-91.

Kobayashi S, Nagino M, Komatsu S, Naruse K, Nimura Y, et al. (2003) Stretch-induced IL-6 secretion from endothelial cells requires NF-kappaB activation. Biochem Biophys Res Commun 308(2): 306-312.

Kool J, Hamdi M, Cornelissen-Steijger P, van der Eb AJ, Terleth C, et al. (2003) Induction of ATF3 by ionizing radiation is mediated via a signaling pathway that includes ATM, Nibrin1, stress-induced MAPkinases and ATF-2. Oncogene 22(27): 4235-4242.

Kosaki K, Ando J, Korenaga R, Kurokawa T, Kamiya A (1998) Fluid shear stress increases the production of granulocyte-macrophage colony-stimulating factor by endothelial cells via mRNA stabilization. Circ Res 82(7): 794-802.

Krebs DL, Hilton DJ (2001) SOCS proteins: negative regulators of cytokine signaling. Stem Cells 19(5): 378-387.

Kuhn MA, Xia G, Mehta VB, Glenn S, Michalsky MP, et al. (2002) Heparin-binding EGF-like growth factor (HB-EGF) decreases oxygen free radical production in vitro and in vivo. Antioxid Redox Signal 4(4): 639-646.

Kunz M, Moeller S, Koczan D, Lorenz P, Wenger RH, et al. (2003) Mechanisms of hypoxic gene regulation of angiogenesis factor Cyr61 in melanoma cells. J Biol Chem 278(46): 45651-45660. Epub 42003 Aug 45625.

Kyriakis JM (2001) Life-or-death decisions. Nature 414(6861): 265-266.

LaBella F, Sive HL, Roeder RG, Heintz N (1988) Cell-cycle regulation of a human histone H2b gene is mediated by the H2b subtype-specific consensus element. Genes Dev 2(1): 32-39.

Lamb JA, Ventura JJ, Hess P, Flavell RA, Davis RJ (2003) JunD mediates survival signaling by the JNK signal transduction pathway. Mol Cell 11(6): 1479-1489.

Lau LF, Lam SC (1999) The CCN family of angiogenic regulators: the integrin connection. Exp Cell Res 248(1): 44-57.

Leone G, Sears R, Huang E, Rempel R, Nuckolls F, et al. (2001) Myc requires distinct E2F activities to induce S phase and apoptosis. Mol Cell 8(1): 105-113.

Leu SJ, Lam SC, Lau LF (2002) Pro-angiogenic activities of CYR61 (CCN1) mediated through integrins alphavbeta3 and alpha6beta1 in human umbilical vein endothelial cells. J Biol Chem 277(48): 46248-46255. Epub 42002 Oct 46242.

Li C, Hu Y, Mayr M, Xu Q (1999) Cyclic strain stress-induced mitogen-activated protein kinase (MAPK) phosphatase 1 expression in vascular smooth muscle cells is regulated by Ras/Rac-MAPK pathways. J Biol Chem 274(36): 25273-25280.

Li C, Zeng Y, Hu J, Yu H (2002) Effects of fluid shear stress on expression of proto-oncogenes c-fos and c-myc in cultured human umbilical vein endothelial cells. Clin Hemorheol Microcirc 26(2): 117-123.

Li CY, Chou TC, Lee CH, Tsai CS, Loh SH, et al. (2003) Adrenaline inhibits lipopolysaccharide-induced macrophage inflammatory protein-1 alpha in human monocytes: the role of beta-adrenergic receptors. Anesth Analg 96(2): 518-523, table of contents.

Li H, Kolluri SK, Gu J, Dawson MI, Cao X, et al. (2000) Cytochrome c release and apoptosis induced by mitochondrial targeting of nuclear orphan receptor TR3. Science 289(5482): 1159-1164.

Liang F, Huang N, Wang B, Chen H (2002a) [Assessment of the role of TLR-4 in shear-stress-induced IL-8 gene transcription activation in vascular endothelial cells by gene mutation and gene transfection technology]. Sheng Wu Yi Xue Gong Cheng Xue Za Zhi 19(4): 667-672.

Liang F, Huang N, Wang B, Chen H, Wu L (2002b) Shear stress induces interleukin-8 mRNA expression and transcriptional activation in human vascular endothelial cells. Chin Med J (Engl) 115(12): 1838-1842.

Liang G, Wolfgang CD, Chen BP, Chen TH, Hai T (1996) ATF3 gene. Genomic organization, promoter, and regulation. J Biol Chem 271(3): 1695-1701.

Liebermann DA, Hoffman B (2002) Myeloid differentiation (MyD)/growth arrest DNA damage (GADD) genes in tumor suppression, immunity and inflammation. Leukemia 16(4): 527-541.

Liebermann DA, Hoffman B (2003) Myeloid differentiation (MyD) primary response genes in hematopoiesis. Blood Cells Mol Dis 31(2): 213-228.

Liu TS, Musch MW, Sugi K, Walsh-Reitz MM, Ropeleski MJ, et al. (2003) Protective role of HSP72 against Clostridium difficile toxin A-induced intestinal epithelial cell dysfunction. Am J Physiol Cell Physiol 284(4): C1073-1082. Epub 2002 Dec 1018.

Long P, Gassner R, Agarwal S (2001) Tumor necrosis factor alpha-dependent proinflammatory gene induction is inhibited by cyclic tensile strain in articular chondrocytes in vitro. Arthritis Rheum 44(10): 2311-2319.

Maira M, Martens C, Philips A, Drouin J (1999) Heterodimerization between members of the Nur subfamily of orphan nuclear receptors as a novel mechanism for gene activation. Mol Cell Biol 19(11): 7549-7557.

Mann DL (2003) Stress-activated cytokines and the heart: from adaptation to maladaptation. Annu Rev Physiol 65: 81-101. Epub 2002 May 2001.

Mannironi C, Orr A, Hatch C, Pilch D, Ivanova V, et al. (1994) The relative expression of human histone H2A genes is similar in different types of proliferating cells. DNA Cell Biol 13(2): 161-170.

Marzluff WF, Gongidi P, Woods KR, Jin J, Maltais LJ (2002) The human and mouse replication-dependent histone genes. Genomics 80(5): 487-498.

McClowry TL, Shors T, Brown DR (2002) Expression of cytokeratin 16 in human papillomavirus type 11-infected genital epithelium. J Med Virol 66(1): 96-101.

Meduri GU (2002) Clinical review: a paradigm shift: the bidirectional effect of inflammation on bacterial growth. Clinical implications for patients with acute respiratory distress syndrome. Crit Care 6(1): 24-29. Epub 2001 Nov 2009.

Miura S, Yamaguchi M, Shimizu N, Abiko Y (2000) Mechanical stress enhances expression and production of plasminogen activator in aging human periodontal ligament cells. Mech Ageing Dev 112(3): 217-231.

Miyazono K, Miyazawa K (2002) Id: a target of BMP signaling. Sci STKE 2002(151): PE40.

Morgan ET, Sewer MB, Iber H, Gonzalez FJ, Lee YH, et al. (1998) Physiological and pathophysiological regulation of cytochrome P450. Drug Metab Dispos 26(12): 1232-1240.

Mysorekar IU, Mulvey MA, Hultgren SJ, Gordon JI (2002) Molecular regulation of urothelial renewal and host defenses during infection with uropathogenic Escherichia coli. J Biol Chem 277(9): 7412-7419. Epub 2001 Dec 7413.

Naka T, Nishimoto N, Kishimoto T (2002) The paradigm of IL-6: from basic science to medicine. Arthritis Res 4(Suppl 3): S233-242. Epub 2002 May 2009.

Nakagomi S, Suzuki Y, Namikawa K, Kiryu-Seo S, Kiyama H (2003) Expression of the activating transcription factor 3 prevents c-Jun N-terminal kinase-induced neuronal death by promoting heat shock protein 27 expression and Akt activation. J Neurosci 23(12): 5187-5196.

Nobes CD, Lauritzen I, Mattei MG, Paris S, Hall A, et al. (1998) A new member of the Rho family, Rnd1, promotes disassembly of actin filament structures and loss of cell adhesion. J Cell Biol 141(1): 187-197.

Nollen EA, Morimoto RI (2002) Chaperoning signaling pathways: molecular chaperones as stress-sensing 'heat shock' proteins. J Cell Sci 115(Pt 14): 2809-2816.

O'Donovan KJ, Tourtellotte WG, Millbrandt J, Baraban JM (1999) The EGR family of transcription-regulatory factors: progress at the interface of molecular and systems neuroscience. Trends Neurosci 22(4): 167-173.

Oh-Hashi K, Maruyama W, Isobe K (2001) Peroxynitrite induces GADD34, 45, and 153 VIA p38 MAPK in human neuroblastoma SH-SY5Y cells. Free Radic Biol Med 30(2): 213-221.

Ohtsuka K, Hata M (2000) Molecular chaperone function of mammalian Hsp70 and Hsp40--a review. Int J Hyperthermia 16(3): 231-245.

Oinuma I, Katoh H, Harada A, Negishi M (2003) Direct interaction of Rnd1 with Plexin-B1 regulates PDZ-RhoGEF-mediated Rho activation by Plexin-B1 and induces cell contraction in COS-7 cells. J Biol Chem 278(28): 25671-25677. Epub 22003 May 25671.

Okamura S, Ng CC, Koyama K, Takei Y, Arakawa H, et al. (1999) Identification of seven genes regulated by wild-type p53 in a colon cancer cell line carrying a well-controlled wild-type p53 expression system. Oncol Res 11(6): 281-285.

Oswald F, Dobner T, Lipp M (1996) The E2F transcription factor activates a replication-dependent human H2A gene in early S phase of the cell cycle. Mol Cell Biol 16(5): 1889-1895.

Oudin S, Pugin J (2002) Role of MAP kinase activation in interleukin-8 production by human BEAS-2B bronchial epithelial cells submitted to cyclic stretch. Am J Respir Cell Mol Biol 27(1): 107-114.

Palmieri EA, Benincasa G, Di Rella F, Casaburi C, Monti MG, et al. (2002) Differential expression of TNF-alpha, IL-6, and IGF-1 by graded mechanical stress in normal rat myocardium. Am J Physiol Heart Circ Physiol 282(3): H926-934.

Pappas P, Stephanou P, Karamanakos P, Vasiliou V, Marselos M (2001) Phenobarbital inducibility and differences in protein expression of an animal model. Chem Biol Interact 130-132(1-3): 275-283.

Paramio JM, Casanova ML, Segrelles C, Mittnacht S, Lane EB, et al. (1999) Modulation of cell proliferation by cytokeratins K10 and K16. Mol Cell Biol 19(4): 3086-3094.

Park HS, Lee JS, Huh SH, Seo JS, Choi EJ (2001) Hsp72 functions as a natural inhibitory protein of c-Jun N-terminal kinase. Embo J 20(3): 446-456.

Park JI, Park HJ, Lee YI, Seo YM, Chun SY (2003) Regulation of NGFI-B expression during the ovulatory process. Mol Cell Endocrinol 202(1-2): 25-29.

Park JM, Adam RM, Peters CA, Guthrie PD, Sun Z, et al. (1999) AP-1 mediates stretch-induced expression of HB-EGF in bladder smooth muscle cells. Am J Physiol 277(2 Pt 1): C294-301.

Pasterkamp RJ, Peschon JJ, Spriggs MK, Kolodkin AL (2003) Semaphorin 7A promotes axon outgrowth through integrins and MAPKs. Nature 424(6947): 398-405.

Pekarsky Y, Hallas C, Palamarchuk A, Koval A, Bullrich F, et al. (2001) Akt phosphorylates and regulates the orphan nuclear receptor Nur77. Proc Natl Acad Sci U S A 98(7): 3690-3694.

Petermann AT, Hiromura K, Blonski M, Pippin J, Monkawa T, et al. (2002) Mechanical stress reduces podocyte proliferation in vitro. Kidney Int 61(1): 40-50.

Pirkkala L, Nykanen P, Sistonen L (2001) Roles of the heat shock transcription factors in regulation of the heat shock response and beyond. Faseb J 15(7): 1118-1131.

Postigo AA, Depp JL, Taylor JJ, Kroll KL (2003) Regulation of Smad signaling through a differential recruitment of coactivators and corepressors by ZEB proteins. Embo J 22(10): 2453-2462.

Prendergast GC (2001) Actin' up: RhoB in cancer and apoptosis. Nat Rev Cancer 1(2): 162-168.

Quentmeier H, Reinhardt J, Zaborski M, Drexler HG (2003) Granulocyte-macrophage colony-stimulating factor: inhibitor of tumor necrosis factor-induced apoptosis. Leuk Res 27(6): 539-545.

Quinn DA, Moufarrej RK, Volokhov A, Hales CA (2002) Interactions of lung stretch, hyperoxia, and MIP-2 production in ventilator-induced lung injury. J Appl Physiol 93(2): 517-525.

Redmond EM, Cahill PA, Hirsch M, Wang YN, Sitzmann JV, et al. (1999) Effect of pulse pressure on vascular smooth muscle cell migration: the role of urokinase and matrix metalloproteinase. Thromb Haemost 81(2): 293-300.

Reibman J, Hsu Y, Chen LC, Kumar A, Su WC, et al. (2002) Size fractions of ambient particulate matter induce granulocyte macrophage colony-stimulating factor in human bronchial epithelial cells by mitogen-activated protein kinase pathways. Am J Respir Cell Mol Biol 27(4): 455-462.

Ridley AJ (2001) Rho proteins: linking signaling with membrane trafficking. Traffic 2(5): 303-310.

Rohan PJ, Davis P, Moskaluk CA, Kearns M, Krutzsch H, et al. (1993) PAC-1: a mitogen-induced nuclear protein tyrosine phosphatase. Science 259(5102): 1763-1766.

Sarkar D, Su ZZ, Lebedeva IV, Sauane M, Gopalkrishnan RV, et al. (2002) mda-7 (IL-24) Mediates selective apoptosis in human melanoma cells by inducing the coordinated overexpression of the GADD family of genes by means of p38 MAPK. Proc Natl Acad Sci U S A 99(15): 10054-10059. Epub 12002 Jul 10011.

Schebesta M, Heavey B, Busslinger M (2002) Transcriptional control of B-cell development. Curr Opin Immunol 14(2): 216-223.

Schlesinger TK, Fanger GR, Yujiri T, Johnson GL (1998) The TAO of MEKK. Front Biosci 3: D1181-1186.

Schwachtgen JL, Houston P, Campbell C, Sukhatme V, Braddock M (1998) Fluid shear stress activation of egr-1 transcription in cultured human endothelial and epithelial cells is mediated via the extracellular signal-related kinase 1/2 mitogen-activated protein kinase pathway. J Clin Invest 101(11): 2540-2549.

Sepulveda AR, Tao H, Carloni E, Sepulveda J, Graham DY, et al. (2002) Screening of gene expression profiles in gastric epithelial cells induced by Helicobacter pylori using microarray analysis. Aliment Pharmacol Ther 16(Suppl 2): 145-157.

Shaulian E, Karin M (2002) AP-1 as a regulator of cell life and death. Nat Cell Biol 4(5): E131-136.

Shi MM, Godleski JJ, Paulauskis JD (1996) Regulation of macrophage inflammatory protein-1alpha mRNA by oxidative stress. J Biol Chem 271(10): 5878-5883.

Sikder HA, Devlin MK, Dunlap S, Ryu B, Alani RM (2003) Id proteins in cell growth and tumorigenesis. Cancer Cell 3(6): 525-530.

Silverman ES, Khachigian LM, Santiago FS, Williams AJ, Lindner V, et al. (1999) Vascular smooth muscle cells express the transcriptional corepressor NAB2 in response to injury. Am J Pathol 155(4): 1311-1317.

Sironen RK, Karjalainen HM, Elo MA, Kaarniranta K, Torronen K, et al. (2002) cDNA array reveals mechanosensitive genes in chondrocytic cells under hydrostatic pressure. Biochim Biophys Acta 1591(1-3): 45-54.

Song KH, Lee K, Choi HS (2002) Endocrine disrupter bisphenol a induces orphan nuclear receptor Nur77 gene expression and steroidogenesis in mouse testicular Leydig cells. Endocrinology 143(6): 2208-2215.

Stefansson S, Lawrence DA (2003) Old dogs and new tricks: proteases, inhibitors, and cell migration. Sci STKE 2003(189): pe24.

Stula M, Orzechowski HD, Gschwend S, Vetter R, von Harsdorf R, et al. (2000) Influence of sustained mechanical stress on Egr-1 mRNA expression in cultured human endothelial cells. Mol Cell Biochem 210(1-2): 101-108.

Suzuki S, Suzuki N, Mirtsos C, Horacek T, Lye E, et al. (2003) Nur77 as a survival factor in tumor necrosis factor signaling. Proc Natl Acad Sci U S A 100(14): 8276-8280. Epub 2003 Jun 8218.

Takahashi H, Tashiro T, Miyazaki M, Kobayashi M, Pollard RB, et al. (2002) An essential role of macrophage inflammatory protein 1alpha/CCL3 on the expression of host's innate immunities against infectious complications. J Leukoc Biol 72(6): 1190-1197.

Tamura I, Rosenbloom J, Macarak E, Chaqour B (2001) Regulation of Cyr61 gene expression by mechanical stretch through multiple signaling pathways. Am J Physiol Cell Physiol 281(5): C1524-1532.

Tarui T, Andronicos N, Czekay RP, Mazar AP, Bdeir K, et al. (2003) Critical role of integrin alpha 5 beta 1 in urokinase (uPA)/urokinase receptor (uPAR, CD87) signaling. J Biol Chem 278(32): 29863-29872. Epub 22003 May 29815.

Tarzami ST, Cheng R, Miao W, Kitsis RN, Berman JW (2002) Chemokine expression in myocardial ischemia: MIP-2 dependent MCP-1 expression protects cardiomyocytes from cell death. J Mol Cell Cardiol 34(2): 209-221.

Tassi E, Al-Attar A, Aigner A, Swift MR, McDonnell K, et al. (2001) Enhancement of fibroblast growth factor (FGF) activity by an FGF-binding protein. J Biol Chem 276(43): 40247-40253. Epub 42001 Aug 40216.

Theodosiou A, Ashworth A (2002) MAP kinase phosphatases. Genome Biol 3(7): REVIEWS3009. Epub 2002 Jun 3026.

Thorpe CM, Smith WE, Hurley BP, Acheson DW (2001) Shiga toxins induce, superinduce, and stabilize a variety of C-X-C chemokine mRNAs in intestinal epithelial cells, resulting in increased chemokine expression. Infect Immun 69(10): 6140-6147.

Tschumperlin DJ, Shively JD, Swartz MA, Silverman ES, Haley KJ, et al. (2002) Bronchial epithelial compression regulates MAP kinase signaling and HB-EGF-like growth factor expression. Am J Physiol Lung Cell Mol Physiol 282(5): L904-911.

Ueda K, Arakawa H, Nakamura Y (2003) Dual-specificity phosphatase 5 (DUSP5) as a direct transcriptional target of tumor suppressor p53. Oncogene 22(36): 5586-5591.

Unoki M, Nakamura Y (2003) EGR2 induces apoptosis in various cancer cell lines by direct transactivation of BNIP3L and BAK. Oncogene 22(14): 2172-2185.

Volloch V, Gabai VL, Rits S, Force T, Sherman MY (2000) HSP72 can protect cells from heat-induced apoptosis by accelerating the inactivation of stress kinase JNK. Cell Stress Chaperones 5(2): 139-147.

Wallasch C, Crabtree JE, Bevec D, Robinson PA, Wagner H, et al. (2002) Helicobacter pylori-stimulated EGF receptor transactivation requires metalloprotease cleavage of HB-EGF. Biochem Biophys Res Commun 295(3): 695-701.

Wang D, Sai J, Richmond A (2003) Cell surface heparan sulfate participates in CXCL1-induced signaling. Biochemistry 42(4): 1071-1077.

Wang N, Planus E, Pouchelet M, Fredberg JJ, Barlovatz-Meimon G (1995) Urokinase receptor mediates mechanical force transfer across the cell surface. Am J Physiol 268(4 Pt 1): C1062-1066.

Watanabe M, Yano W, Kondo S, Hattori Y, Yamada N, et al. (2003) Up-regulation of urokinase-type plasminogen activator in corneal epithelial cells induced by wounding. Invest Ophthalmol Vis Sci 44(8): 3332-3338.

Wennerberg K, Forget MA, Ellerbroek SM, Arthur WT, Burridge K, et al. (2003) Rnd proteins function as RhoA antagonists by activating p190 RhoGAP. Curr Biol 13(13): 1106-1115.

Xia G, Martin AE, Besner GE (2003) Heparin-binding EGF-like growth factor downregulates expression of adhesion molecules and infiltration of inflammatory cells after intestinal ischemia/reperfusion injury. J Pediatr Surg 38(3): 434-439.

Yamamoto H, Teramoto H, Uetani K, Igawa K, Shimizu E (2002) Cyclic stretch upregulates interleukin-8 and transforming growth factor-beta1 production through a protein kinase C-dependent pathway in alveolar epithelial cells. Respirology 7(2): 103-109.

Yang SK, Eckmann L, Panja A, Kagnoff MF (1997) Differential and regulated expression of C-X-C, C-C, and C-chemokines by human colon epithelial cells. Gastroenterology 113(4): 1214-1223.

Yasukawa H, Sasaki A, Yoshimura A (2000) Negative regulation of cytokine signaling pathways. Annu Rev Immunol 18: 143-164.

Yasukawa H, Hoshijima M, Gu Y, Nakamura T, Pradervand S, et al. (2001) Suppressor of cytokine signaling-3 is a biomechanical stress-inducible gene that suppresses gp130-mediated cardiac myocyte hypertrophy and survival pathways. J Clin Invest 108(10): 1459-1467.

Yin Y, Liu YX, Jin YJ, Hall EJ, Barrett JC (2003) PAC1 phosphatase is a transcription target of p53 in signalling apoptosis and growth suppression. Nature 422(6931): 527-531.

Zalcman G, Closson V, Linares-Cruz G, Lerebours F, Honore N, et al. (1995) Regulation of Ras-related RhoB protein expression during the cell cycle. Oncogene 10(10): 1935-1945.

Zangerl B, Zhang Q, Pearce-Kelling SE, Aguirre GD (2002) Molecular cloning, characterization and mapping of the canine glucocorticoid receptor DNA binding factor 1 (GRLF1). Gene 294(1-2): 167-176.

Zeng XR, Sun Y, Wenger L, Cheung HS (2003) Induction of early growth response gene Egr2 by basic calcium phosphate crystals through a calcium-dependent protein kinase C-independent p44/42 mitogen-activated protein kinase pathway. Cells Tissues Organs 174(1-2): 63-72.

Zhu Y, Jin K, Mao XO, Greenberg DA (2003) Vascular endothelial growth factor promotes proliferation of cortical neuron precursors by regulating E2F expression. Faseb J 17(2): 186-193.
